# Efficacy of Dang Gui Shao Yao San in treating vascular dementia in animal models: a systematic review and meta-analysis

**DOI:** 10.3389/fnagi.2026.1701536

**Published:** 2026-03-13

**Authors:** Gaoxuan Qu, Yifan Bi, Lingzhi Wang, Li Xu, Yan Zhang, Yan Ma, Caijun Tian, Zhe Zhang

**Affiliations:** 1 The First Clinical Medical College, Shandong University of Traditional Chinese Medicine, Jinan, China; 2 Faculty of Chinese Medicine, Macao University of Science and Technology, Macao, China; 3 College of Foreign Language, Shandong University of Traditional Chinese Medicine, Jinan, China; 4 College of Traditional Chinese Medicine, Shandong University of Traditional Chinese Medicine, Jinan, China; 5 College of Pharmacy, Shandong University of Traditional Chinese Medicine, Jinan, China

**Keywords:** animal models, Dang Gui Shao Yao San, neuroprotection, oxidative stress, traditional Chinese medicine, vascular dementia

## Abstract

**Background:**

Vascular dementia (VaD) is the second most prevalent form of dementia, following Alzheimer’s disease (AD), and severely impacts life quality of patients. Currently, effective strategies to alleviate symptoms and delay disease progression remain unavailable. Animal studies indicate that Dang Gui Shao Yao San (DGSYS) may enhance cognitive function in VaD models through mechanisms such as antioxidation and the enhancement of cerebral blood flow. However, a systematic review of its neuroprotective effects and a comprehensive assessment of its translational potential in preclinical models are still lacking.

**Objective:**

This study seeks to synthesize evidence on the intervention effects and underlying mechanisms of DGSYS in animal models of VaD through a systematic review and meta-analysis, so as to offer preclinical evidence to inform future research.

**Methods:**

A comprehensive literature search was performed across eight databases: China National Knowledge Infrastructure (CNKI), VIP Database for Chinese Technical Periodicals, Wanfang Data, SinoMed, Web of Science, Cochrane Library, PubMed, and EMBASE, to identify studies published from the inception to May 2025 on the effects of DGSYS in animal models of VaD. The primary outcomes were cognitive and behavioral assessments, histopathological alterations in brain tissue, and biomarkers of oxidative stress. The quality of the included studies was assessed using a 10-item checklist, and meta-analysis was conducted with RevMan 5.4 software.

**Results:**

Seven studies involving 420 rats and mice were included. DGSYS notably enhanced cognitive and behavioral performance in the animal models. Specifically, DGSYS significantly improved spatial learning and memory performance, as assessed by the Morris water maze, and attenuated oxidative stress–related pathological changes.

**Conclusion:**

Current animal studies provide evidence for the multidimensional neuroprotective effects of DGSYS in VaD, with potential mechanisms involving antioxidative stress, inhibition of neuroinflammation, and enhancement of cerebral microcirculation. However, substantial heterogeneity across outcome measures and generally low methodological quality were observed. Overall, the available evidence suggests potential neuroprotective effects of DGSYS in preclinical models, warranting further standardized and rigorously designed studies before clinical translation can be considered.

**Systematic review registration:**

https://www.crd.york.ac.uk/PROSPERO/view/CRD420251058443, identifier CRD420251058443.

## 1 Introduction

Vascular dementia (VaD) is a progressive disorder resulting from vascular brain injury, typically involving parenchymal brain damage caused by ischemia, infarction, or hemorrhage (Chang Wong and Chang Chui, 2022). As the second most prevalent form of dementia after Alzheimer’s disease (AD), VaD has long represented a major issue in global public health (Iadecola et al., 2019). VaD significantly impacts affected individuals and imposes substantial social and economic burdens.

Epidemiologically, VaD is generally considered a leading non-AD dementia subtype and is often reported to represent a substantial proportion of dementia cases, although estimates vary across populations and diagnostic approaches (Tuominen et al., 2025). From an etiological perspective, VaD arises from cognitive impairment attributable to cerebrovascular pathology. The vascular substrates implicated in VaD commonly include large-vessel and strategic infarcts, as well as cerebral small vessel disease–related lesions such as lacunes, microinfarcts, and diffuse white-matter changes (Kalaria, 2018; Inoue et al., 2023).

Building on these lesion patterns, a growing body of evidence indicates that VaD pathophysiology is characterized by chronic cerebral hypoperfusion and blood–brain barrier dysfunction, which subsequently initiate downstream cellular stress responses, including neuroinflammation, apoptosis, and dysregulated autophagy, ultimately contributing to neuronal injury and cognitive decline (Iadecola, 2013; Venkat et al., 2015; Wang et al., 2020).

Currently, pharmacological treatment options for VaD and their efficacy are still somewhat limited (Tisher and Salardini, 2019). Clinically used agents for vascular dementia, including memantine and cholinesterase inhibitors, are primarily symptomatic and show at most modest or inconsistent cognitive benefits, while adverse effects such as dizziness, insomnia, hallucinations, gastrointestinal disturbances, and occasional worsening cognition may limit long-term use (Dunn et al., 2000; Lipton, 2004; Kavirajan and Schneider, 2007; McShane et al., 2019). Therefore, there is an urgent need to identify alternative therapeutic agents for VaD.

Biomarker research in VaD has increasingly focused on fluid indicators of blood–brain barrier disruption and vascular injury. Reported candidates include the CSF/serum albumin quotient, von Willebrand factor, thrombomodulin, and myelin basic protein (Hagnelius et al., 2010; Cipollini et al., 2019; Musaeus et al., 2020; Hosoki et al., 2021). Tight junction–related proteins such as occludin and claudin-5 have also been explored as circulating readouts of barrier integrity in cerebrovascular disease and vascular cognitive disorders that overlap mechanistically with VaD (Lasek-Bal et al., 2020; Hansra et al., 2024). Overall, these biomarkers highlight the vascular and barrier-related substrate of VaD and provide mechanistic context for translational research and endpoint selection. Importantly, this mechanistic framework also supports interest in multi-target therapeutic strategies that can engage several convergent pathways and can be evaluated using clinically relevant, mechanism-linked outcomes. However, these biomarkers were not assessed in the experimental studies included in the present meta-analysis and are therefore discussed here only as contextual background rather than analyzed outcomes.

Recent reviews emphasize that dementia-related cognitive disorders are driven by multifactorial and multi-pathway processes, which has sustained interest in multi-target strategies, including natural products and traditional medicinal plants (Tripathi et al., 2024). In parallel, recent systematic reviews and meta-analyses have evaluated several TCM-related interventions for VaD or vascular cognitive disorders, including *Bu Yang Huan Wu Tang* in patients (Kim et al., 2022) and acupuncture-based interventions in VaD models (Li G. et al., 2022; Bao et al., 2024), underscoring the rapidly evolving evidence landscape. Moreover, advances in multi-target drug-design approaches further underscore the need for a rigorous, systematic synthesis of mechanistically grounded preclinical evidence to support translation (Shrivastava et al., 2022). As a key component of traditional Chinese medicine (TCM), Chinese medicinal remedies offer multi-target and multi-pathway regulation with fewer side effects, showing great potential in the treatment of VaD (Li et al., 2021; Tao et al., 2022).

*Dang Gui Shao Yao San* (DGSYS) is a TCM formula derived from *Synopsis of the Golden Chamber* written by Zhang Zhongjing in Han Dynasty. It consists of six Chinese medicinals: *Angelica sinensis* (*Dang Gui*), *Paeonia lactiflora* (*Shao Yao*), *Poria cocos* (*Fu Ling*), *Alisma orientale* (*Ze Xie*), *Ligusticum chuanxiong* (*Chuan Xiong*), and *Atractylodes macrocephala* (*Bai Zhu*). DGSYS has been evaluated in clinical studies of dementia and has been summarized in a systematic review of human studies (Kim and Cho, 2020), including studies in vascular dementia. However, the available clinical evidence remains limited and does not address key mechanistic outcomes and internal validity issues that are commonly assessed in animal models. Phytochemical analyses have identified multiple potentially bioactive constituents in DGSYS, including paeoniflorin and ferulic acid (Chen et al., 2009). However, the present review focuses on efficacy outcomes and mechanism-related evidence reported in VaD animal studies rather than compound-level pharmacology.

Although a growing number of animal studies have investigated the therapeutic effects of DGSYS in the context of VaD research, a comprehensive meta-analysis integrating and quantitatively assessing this evidence is still lacking. Moreover, while several reviews have discussed Chinese medicinal therapies for VaD in general, no systematic meta-analysis has specifically focused on DGSYS. Importantly, we focused exclusively on DGSYS because pooling different multi-herb formulae would introduce substantial formulation-, dose-, and indication-related heterogeneity, potentially obscuring formula-specific effects. In contrast, DGSYS represents a well-defined prescription that has been repeatedly tested in VaD-relevant animal models, enabling quantitative synthesis across comparable cognitive and mechanistic outcomes. Therefore, this study addresses an important research gap, as preclinical studies of DGSYS in VaD models have not been systematically synthesized or quantitatively pooled, and the methodological quality of the available evidence has not been comprehensively appraised.

Previous reviews and meta-analyses have summarized TCM and Chinese herbal medicine for VaD and related cognitive disorders, mainly at the level of broad formulae and mixed interventions rather than a single, well-defined formula (Xu et al., 2018; Bai and Zhang, 2021). It should be noted that many multi-component traditional formulations have been investigated in dementia models (Kun et al., 2025); however, these formulations differ substantially in composition and therapeutic focus, making direct comparisons difficult. In contrast, DGSYS represents a defined formula that has been repeatedly evaluated in VaD-relevant models and shows a relatively consistent mechanistic pattern related to neurovascular dysfunction and ischemia-associated pathways.

Consequently, the evidence specific to DGSYS, which spans efficacy outcomes, methodological quality, and mechanistic rationale, remains fragmented and has not been systematically appraised. Therefore, this systematic review aims to comprehensively synthesize the available preclinical evidence on DGSYS in VaD, focusing on cognitive outcomes and key mechanistic pathways including modulation of neurotransmitter systems, attenuation of neuroinflammatory responses, and inhibition of neuronal apoptosis. By integrating findings across study types, we seek to clarify the strength and limitations of current evidence and to inform future translational and clinical research on DGSYS.

## 2 Methods

This meta-analysis was registered in the International Prospective Register of Systematic Reviews (PROSPERO) on 11 June, 2025 (CRD420251058443).

### 2.1 Literature search

VIP Journal Integration Platform (VJIP), China National Knowledge Infrastructure (CNKI), Wanfang Data, SinoMed, Web of Science, PubMed, Cochrane Library, and EMBASE were searched to identify animal studies on the use of DGSYS for treating VaD published from the inception to April 26, 2025. We included studies published in Chinese or English. For the Chinese databases, search terms included “vascular dementia” (in Chinese) and “dementia” (in Chinese), while those related to DGSYS included “Dang Gui Shao Yao San” (in Chinese), “modified Dang Gui Shao Yao San” (in Chinese) and “Jia Wei Dang Gui Shao Yao San” (in Chinese). The CNKI search strategy was as follows: Topic Keyword Approach (TKA) = [“Dang Gui Shao Yao San” (in Chinese) + “modified Dang Gui Shao Yao San” (in Chinese) + “Jia Wei Dang Gui Shao Yao San” (in Chinese)] AND TKA = [“vascular dementia” (in Chinese) + “dementia” (in Chinese)] AND Full Text (FT) = [“rat” (in Chinese) + “mouse” (in Chinese)]. For the English databases, the key words included “vascular dementia” and “Dang Gui Shao Yao San,” with the search strategy using both subject headings and free-text terms. Cross-database searches were performed to ensure comprehensive coverage and prevent omissions. The search strategy used in each database is shown in Supplementary Files.

### 2.2 Inclusion criteria

Rodent models were used as study subjects, regardless of the methods used to induce VaD. The intervention was primarily based on DGSYS, with no restrictions on dosage, administration, or treatment duration. Primary outcome measures included performance in the Morris water maze (MWM) test, neuropathological changes in the hippocampus, markers of apoptosis, and indicators of oxidative stress. Studies must comply with animal ethical standards, ensuring the welfare of laboratory animals.

### 2.3 Exclusion criteria

Non-rodent animal models. Studies without a DGSYS treatment group, regardless of whether it was used alone or in combination with other interventions. Studies lacking data on the MWM, hippocampal pathology, apoptosis markers, or oxidative stress indicators. *In vitro* studies or cell-based experiments without animal data. Studies that did not report the number of animals used in experiments. Studies were excluded a priori if anaesthesia/analgesia procedures raised substantial animal-welfare and methodological concerns (e.g., use of chloral hydrate) or if essential details of anaesthesia/analgesia were not reported. Reporting of anaesthesia/analgesia was evaluated in line with ARRIVE 2.0 recommendations (Percie du Sert et al., 2020), and ethical oversight was considered with reference to institutional animal care and use standards.

### 2.4 Data extraction

All retrieved references were imported into NoteExpress reference management software (version 4.1.0, Aegean Software Corporation, Beijing, China) to identify and remove the duplicated papers automatically. The remaining studies were independently screened in full text by two reviewers according to the predefined inclusion and exclusion criteria. In cases of disagreement, a third reviewer was consulted to make the final decision. The screening results were then cross-checked for consistency by both reviewers. Inter-rater agreement for full-text eligibility decisions was quantified using Cohen’s κ coefficient. Relevant data were extracted from the included studies.

For continuous outcomes, we extracted the sample size, mean, and standard deviation for each group. When studies reported medians with interquartile ranges, we converted these data to means and standard deviations using established methods (McGrath et al., 2020). When the estimated standard deviation was zero, the study was not entered into the meta-analysis dataset for that outcome and was summarized narratively.

When a study reported multiple DGSYS dose arms, we extracted data from the highest-dose arm for the primary meta-analyses to avoid unit-of-analysis issues arising from multiple comparisons within the same study. We acknowledge that “high dose” was not standardized across experiments and that dosing units/regimens varied substantially, which precluded meaningful cross-study dose normalization or pooled dose–response modeling. Therefore, this approach may overestimate efficacy and is interpreted cautiously; we report it explicitly as a limitation. For outcome data presented graphically, attempts were made to contact the original authors to obtain precise numerical values. If no response was received, WebPlotDigitizer (version 4.7, Ankit Rohatgi)^1^ was used to estimate the data.

### 2.5 Quality assessment and risk of bias evaluation

Quality assessment was performed using a 10-item checklist tool (Sena et al., 2007). This checklist is commonly used in preclinical systematic reviews to capture key design and reporting features. Each item was rated as “Yes,” “No,” or “Unclear,” with “Yes” assigned a score of 1 and the others scored 0. The total quality score for each study was calculated. In addition, risk of bias was assessed using SYRCLE’s Risk of Bias tool for animal intervention studies, and judgments were summarized as low, high, or unclear risk of bias (Hooijmans et al., 2014). Two reviewers independently assessed the study quality, and discrepancies were resolved through discussion with a third reviewer.

### 2.6 Certainty of the evidence

The certainty of evidence for each main outcome was assessed using the GRADE approach adapted for preclinical animal intervention studies (Hooijmans et al., 2018). We considered the domains of risk of bias, inconsistency, indirectness, imprecision, and publication bias. Risk-of-bias judgments were informed by the SYRCLE tool. Inconsistency was evaluated based on between-study heterogeneity and the consistency of effect direction. Indirectness was judged by the relevance of the animal model and outcome measures to VaD. Imprecision was judged by sample size and confidence interval width. Publication bias was assessed using funnel plots only when at least 10 independent studies were available for an outcome; otherwise, it was not formally assessed due to limited power.

### 2.7 Statistical analysis

Statistical analyses were conducted using RevMan 5.4 software. We used a random-effects model for all meta-analyses because between-study heterogeneity was expected across animal species, VaD models, dosing regimens, and outcome measures. Statistical heterogeneity was quantified using the Chi-square test and the *I*^2^ statistic. The choice of meta-analysis model was not based on heterogeneity tests (Higgins et al., 2024). In cases of significant heterogeneity, subgroup analyses or sensitivity analyses were performed to assess the stability of the results.

For continuous outcomes, we used mean difference (MD) with 95% confidence intervals when studies reported the outcome on an identical scale and unit. This applied to MWM escape latency reported in seconds, the number of platform crossings, superoxide dismutase (SOD), and malondialdehyde (MDA). Studies were pooled only when a usable measure of variability was available. When variability data were unavailable or the standard deviation was zero, the study was excluded from the meta-analysis for that outcome and was summarized narratively.

When outcomes reflected the same construct but were reported under different scales or instruments, we used standardized mean difference (SMD). Where direct comparability was met, we explored heterogeneity through prespecified subgroup and sensitivity analyses. We explored between-study heterogeneity through prespecified subgroup analyses and sensitivity analyses. Prediction intervals were not calculated for subgroups containing fewer than three studies, as reliable estimation requires “at least three studies” and often yields uninformatively “very wide” intervals given limited data (Riley et al., 2011). A *P-*value ≤ 0.05 was considered statistically significant.

## 3 Results

### 3.1 Study inclusion

**FIGURE 1:**
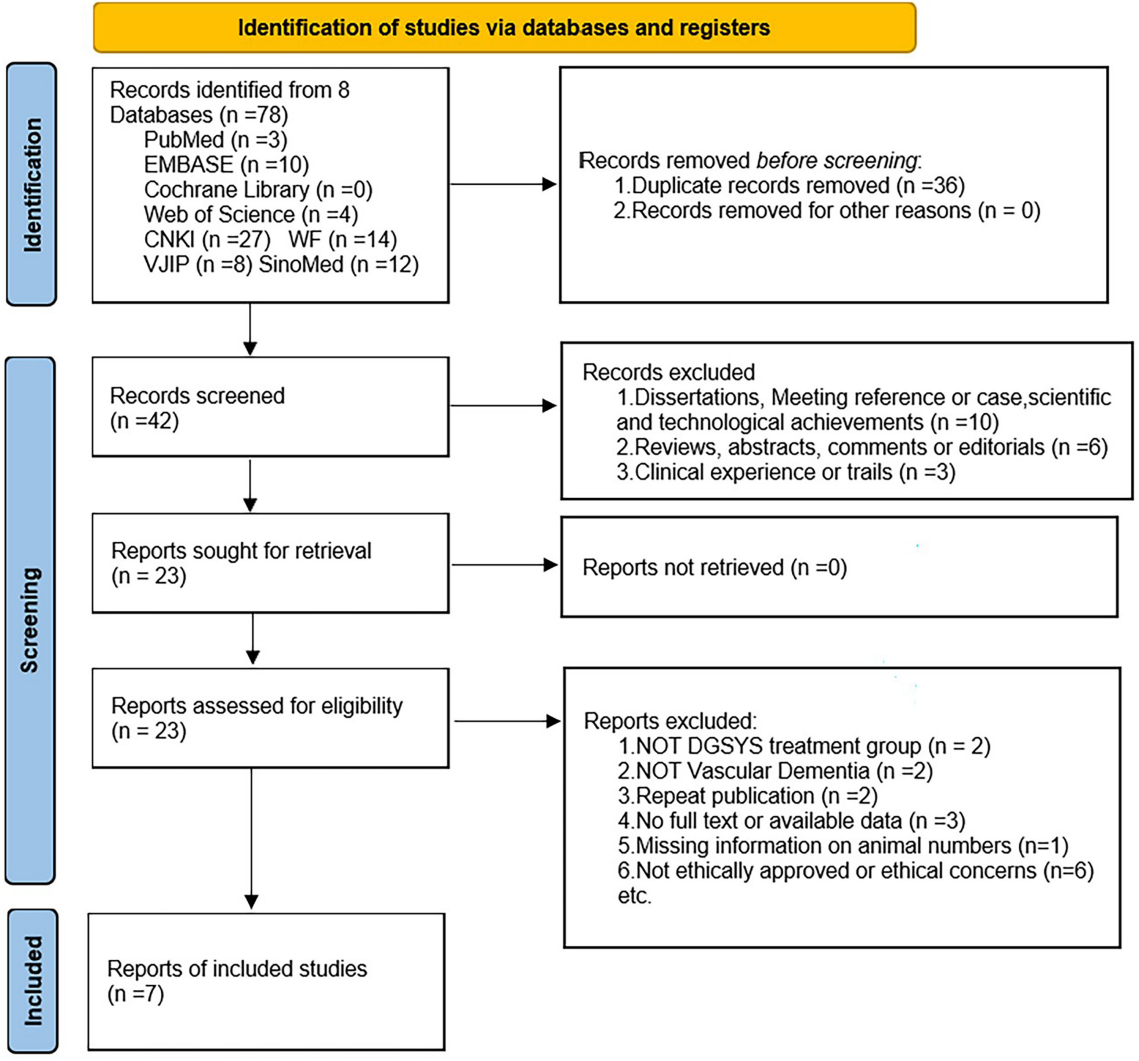
PRISMA 2020 flow diagram of the study selection process (adapted from Page et al., 2021).

A total of 78 studies were initially identified through the database search. After 36 duplicate records were removed, forty-two studies remained. After screening titles and abstracts, 19 studies were excluded because they are case reports, conference papers, dissertations, research briefs, clinical studies, reviews, or systematic reviews/meta-analyses that did not meet the inclusion criteria. A full-text screening was then conducted on the remaining studies. An additional 16 studies were excluded for the following reasons: use of non-rodent models, absence of a DGSYS treatment group, lack of relevant outcome data, *in vitro* study design, missing information on animal numbers, or the use of unethical practices such as chloral hydrate anesthesia. Ultimately, 7 studies were included in the final analysis. The searching process is shown in Figure 1. Data extraction was conducted independently by two reviewers, yielding high agreement (Cohen’s κ = 0.76).

### 3.2 Study characteristics

The included studies were published between 2003 and 2025, with four articles published in English. Among the 7 studies, bilateral common carotid artery occlusion (BCCAO) was employed in 5 studies to establish VaD models, bilateral carotid artery stenosis (BCAS) was used in 1 study, and simple ischemia-reperfusion methods was used in 1 study. A total of 420 rats or mice were involved, including 290 rats and 130 mice, with sample sizes ranging from 50 to 70 per study. Three different rodent strains were used across studies: Sprague-Dawley (SD) rats, Kunming mice, and C57BL/6J mice. Six studies used male rodents and one study used female rodents exclusively. Study characteristics are shown in Table 1.

**TABLE 1:** Characteristics of the included studies.

Study (first author, year)	Animal species, gender, weight	Sample sizes	Modeling method	Anesthetics	Experimental group	Control group
Ningning et al. (2024)	Male SD rats, 250–280 g	50	BCCAO	Urethane	Low dose of DGSYS group (L-DGSYS, 5 g/kg), high dose of DGSYS group (H-DGSYS, 10 g/kg)	Sham group, model group, nimodipine (9.45 mg/kg) group
Cai et al. (2020)	Female SD rats, 250–300 g	60	BCCAO	Pentobarbital sodium	Low-dose DGSYS group (L-DGSYS, 1.8 g/kg/d), high-dose DGSYS group (H-DGSYS, 7.2 g/kg/d)	Sham group, model group, nimodipine (20 mg/kg) group
Bin et al. (2022)	Male SD rats, 250–280 g	60*	BCCAO	Pentobarbital sodium	Low-dose DGSYS group (L-DGSYS, 1.6 g/kg/d), high-dose DGSYS group (H-DGSYS, 6.4 g/kg/d)	Sham group, model group, nimodipine (9.45 mg/kg) group
Meijie et al. (2024)	Male SD rats, 180–220 g	60	BCCAO	Pentobarbital sodium	Low-dose DGSYS group (L-DGSYS, 1.8 g/kg/d), high-dose DGSYS group (H-DGSYS, 7.2 g/kg/d)	Sham group, model group, nimodipine (1.68 g/kg/d) group
Huiyuan et al. (2003)	Male kunming mice, 20–22 g	60	Mouse memory impairment induced by repeated ischemia-reperfusion	Urethane	DGSYS group (0.3 mL/d)	Normal control group, sham group, model group
Chuyao et al. (2025)	Male C57BL/6J mice, 20–22 g	70	BCAS	Isoflurane	Low-dose DGSYS group (L-DGSYS, 12 g/kg), middle-dose DGSYS group (M-DGSYS, 24 g/kg), high-dose DGSYS group (H-DGSYS, 36 g/kg)	Normal control group, sham group, model group, nimodipine (10 mg/kg/d) group
Yixiu et al. (2025)	Male SD rats, 180–220 g	60	BCCAO	Not mentioned	Low-dose DGSYS group (L-DGSYS, 1.8 g/kg/d), High-dose DGSYS group (H-DGSYS, 7.2 g/kg/d)	Sham group, model group, nimodipine (20 mg/kg/d) group

BCCAO, bilateral common carotid arteries occlusion; BCAS, bilateral common carotid artery stenosis; DGSYS, Dang Gui Shao Yao San. * Sixty rats were initially enrolled; 10 died during the experiment; 50 rats were included in the final analyses.

Regarding anesthesia, urethane was used in 2 studies, pentobarbital sodium in 3 studies, and isoflurane in 1 study; however, in 1 study the anesthetic agent used was not specified. In terms of study design, 6 studies included multiple DGSYS dosage groups and a positive drug control group. Five studies included both a sham-operated group and a model group as controls, while two studies additionally incorporated a blank control group.

In 7 studies MWM was employed to assess learning and memory outcomes. In 5 studies neuronal morphology in the hippocampus was examined, in 2 studies TNF-α expression was assessed, in 3 studies Bcl-2 and Bax protein levels was examined, and in 2 studies, oxidative stress indicators such as SOD, MDA, and reactive oxygen species (ROS) were measured.

### 3.3 Quality assessment and risk of bias evaluation

The quality scores of the 7 included studies ranged from 4 to 6, with an average score of 5.143. All studies were published in peer-reviewed journals and clearly reported the animal species used, and employed random allocation. In 5 studies temperature control during the experiments was conducted, and in 3 studies anesthetic agents without intrinsic neuroprotective properties were used. In 6 studies compliance with animal welfare regulations was explicitly stated, and in 2 studies no potential conflicts of interest were declared. However, in all the studies sample size calculations were not reported, neither the use of blinding during model establishment or outcome assessment. The quality assessment of the included studies is shown in Table 2.

**TABLE 2:** Quality assessment of the included studies.

Study (first author, year)	Publication in peer-reviewed journal	Temperature control	Random allocation to treatment/control group	Blinded model induction	Blinded outcome assessment	Anesthetic without intrinsic neuroprotective activity	Stated animal species	Sample size calculation	Compliance with animal welfare regulations	Declaration of conflicts of interest	Score
Ningning et al. (2024)	Yes	No	Yes	No	No	Yes	Yes	No	Yes	Yes	6
Cai et al. (2020)	Yes	Yes	Yes	No	No	No	Yes	No	Yes	Yes	6
Bin et al. (2022)	Yes	Yes	Yes	No	No	No	Yes	No	Yes	No	5
Meijie et al. (2024)	Yes	Yes	Yes	No	No	No	Yes	No	Yes	No	5
Huiyuan et al. (2003)	Yes	Yes	Yes	No	No	Yes	Yes	No	Unclear	No	5
Chuyao et al. (2025)	Yes	Yes	Yes	No	No	Yes	Yes	No	Yes	No	5
Yixiu et al. (2025)	Yes	No	Yes	No	No	Unclear	Yes	No	Yes	No	4

Risk of bias was generally unclear due to incomplete reporting. Although several studies stated that animals were randomly allocated, the method used to generate the random sequence was often not specified, and allocation concealment was rarely described. Random housing and blinding procedures for caregivers, investigators, and outcome assessors were frequently not reported, resulting in predominantly unclear risk ratings in the performance and detection domains. In addition, some studies provided insufficient information on exclusions and attrition, leaving the risk related to incomplete outcome data unclear. Overall, these issues indicate that the risk of bias in the included studies cannot be confidently ruled out, and the findings should be interpreted with caution. The SYRCLE risk-of-bias assessment results are presented in Table 3.

**TABLE 3:** SYRCLE risk-of-bias assessment for included animal studies.

Study (first author, year)	Sequence generation	Baseline characteristics	Allocation concealment	Random housing	Blinding caregivers/ investigators	Random outcome assessment	Blinding outcome assessor	Incomplete outcome data	Selective outcome reporting	Other bias
Ningning et al. (2024)	Unclear	Unclear	Unclear	Unclear	Unclear	Unclear	Unclear	Unclear	Unclear	Unclear
Cai et al. (2020)	Unclear	Unclear	Unclear	Unclear	Unclear	Unclear	Unclear	Low	Unclear	Unclear
Bin et al. (2022)	Low	Unclear	Unclear	Unclear	Unclear	Unclear	Unclear	Unclear	Unclear	Unclear
Meijie et al. (2024)	Unclear	Unclear	Unclear	Unclear	Unclear	Unclear	Unclear	Unclear	Unclear	Unclear
Huiyuan et al. (2003)	Unclear	Unclear	Unclear	Unclear	Unclear	Unclear	Unclear	Unclear	Unclear	Unclear
Chuyao et al. (2025)	Unclear	Unclear	Unclear	Unclear	Unclear	Unclear	Unclear	Unclear	Unclear	Unclear
Yixiu et al. (2025)	Unclear	Unclear	Unclear	Unclear	Unclear	Unclear	Unclear	Unclear	Unclear	Unclear

### 3.4 Certainty of the evidence

We assessed the certainty of evidence for each outcome using the GRADE approach adapted for preclinical studies. Overall, the certainty was moderate for most outcomes, including MWM performance, tumor necrosis factor alpha, superoxide dismutase, reactive oxygen species, and malondialdehyde. This was mainly due to serious risk of bias arising from insufficient reporting of key methodological safeguards such as blinding and randomization, which led to a one level downgrade from the initial high rating. In contrast, the certainty was low for hippocampal neuronal morphology and the Bcl-2/Bax ratio because of combined downgrades for risk of bias and serious imprecision, driven by limited quantitative reporting for morphology and wide confidence intervals that crossed the null for the Bcl-2/Bax ratio. No downgrades were applied for inconsistency or indirectness. The results for the certainty of the evidence are presented in Table 4.

**TABLE 4:** GRADE evidence certainty worksheet.

Outcome (definition/timepoint; direction of benefit)	Priority (critical/important)	Studies (n)	Animals (n) (or experimental units)	Effect estimate (95% CI) (RR/OR/MD/SMD/HR)	Starting certainty (high/moderate/low)	Risk of bias (downgrade)	Inconsistency (downgrade)	
MWM escape latency (lower is better)	Critical	6	126	MD = –27.21, 95% CI [-30.36, -24.05]	High	Serious	None	
MWM platform crossings (higher is better)	Critical	5	104	MD = 2.61, 95% CI [1.70, 3.52]	High	Serious	None	
Hippocampal neuronal morphology	Important	5	88	Improvement reported in 5 studies	High	Serious	None	
TNF-α Expression (lower is better)	Critical	2	40	SMD = –4.11, 95% CI [–9.76, 1.53]	High	Serious	None	
Bcl-2/Bax ratio (higher is better)	Important	3	60	SMD = –0.35, 95% CI [–4.30, 3.59]	High	Serious	None	
SOD (higher is better)	Critical	2	44	MD = 35.52, 95% CI [15.16, 55.88]	High	Serious	None	
ROS (lower is better)	Critical	2	44	SMD = –2.63, 95% CI [–5.21, –0.04]	High	Serious	None	
MDA (lower is better)	Critical	2	44	MD = –2.56, 95% CI [–4.40, –0.72]	High	Serious	None	
**Indirectness L1: Preclinical PICO match (downgrade)**	**Indirectness L2: translatability to clinical PICO (downgrade)**	**Imprecision (downgrade)**	**Publication bias (downgrade)**	**Upgrade: large effect (0–2)**	**Upgrade: dose–response (0–1)**	**Upgrade: residual confounding (0–1)**	**Final certainty (high/moderate/low/very low)**	**Notes/rationale (1–2 sentences per key decision)**
None	None	None	None	0	0	0	Moderate	Most studies did not report blinding; key risk-of-bias domains were unclear.
None	None	None	None	0	0	0	Moderate	Most studies did not report blinding; key risk-of-bias domains were unclear.
None	None	Serious	Suspected	0	0	0	Low	Only representative images were presented without a described sampling strategy.
None	None	None	None	0	0	0	Moderate	Most studies did not report blinding; key risk-of-bias domains were unclear.
None	None	Serious	None	0	0	0	Low	Most studies did not report blinding; 95% CI crossed the null (imprecision).
None	None	None	None	0	0	0	Moderate	Most studies did not report blinding; key risk-of-bias domains were unclear.
None	None	None	None	0	0	0	Moderate	Most studies did not report blinding; key risk-of-bias domains were unclear.
None	None	None	None	0	0	0	Moderate	Most studies did not report blinding; key risk-of-bias domains were unclear.

### 3.5 Effect of DGSYS on behavioral performance

**FIGURE 2:**
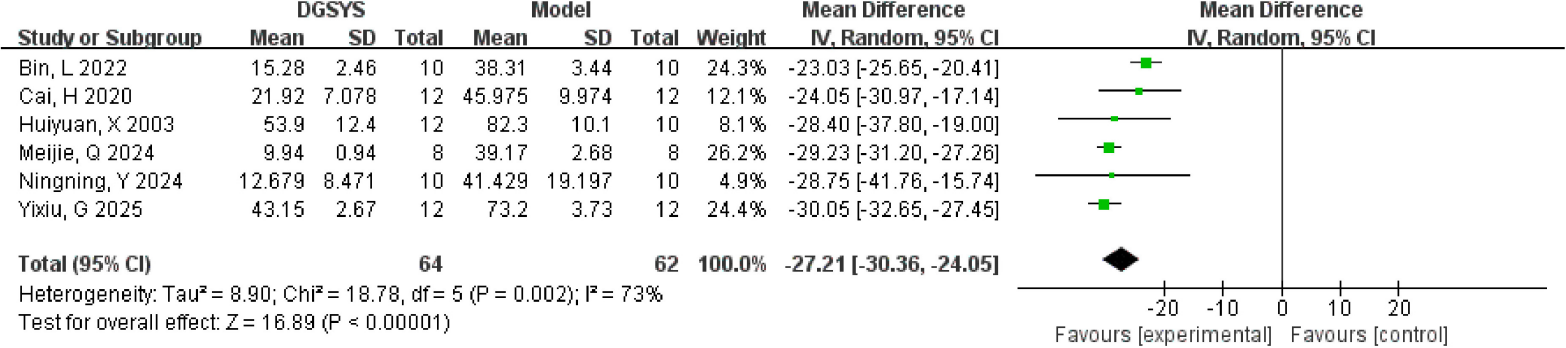
Forest plot of escape latency in the MWM test (DGSYS vs. control). Random-effects meta-analysis of 6 studies showed that DGSYS significantly reduced escape latency compared with control (nT/nC = 64/62, MD = –27.21, 95% CI: –30.36 to –24.05, *P* < 0.00001; heterogeneity τ^2^ = 8.90, χ^2^ = 18.78, df = 5, *P* = 0.002, *I*^2^ = 73%). Effect measure: MD (original units); lower values indicate better performance.

In 6 studies the MWM test was employed, encompassing both the place navigation (acquisition) and spatial probe (retention) trials. In the 6 studies, escape latency was extracted from the place navigation trial as an outcome measure. Of these, in 4 studies data were expressed in numerical form, while in 2 studies data were in graphical form, from which values were estimated using digital ruler software (WebPlotDigitizer version 4.7, Ankit Rohatgi) (see text footnote 1). According to the meta-analysis of the 6 studies, it was revealed that DGSYS significantly reduced escape latency compared to the control group [*n* = 126, MD = –27.21, 95% CI (–30.36, –24.05), *P* < 0.0001]. However, substantial heterogeneity was observed across studies (Tau^2^ = 8.90, df = 5, *P* = 0.002; *I*^2^ = 73%) as shown in Figure 2.

**FIGURE 3:**
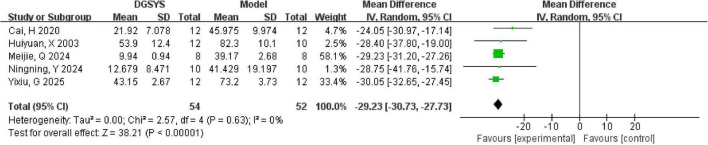
Forest plot of escape latency in the MWM test (DGSYS vs. control). Random-effects meta-analysis of 5 studies showed that DGSYS significantly reduced escape latency compared with control (nT/nC = 54/52, MD = –29.23, 95% CI: –30.73 to –27.73, *P* < 0.00001; heterogeneity τ^2^ = 0.00, χ^2^ = 2.57, df = 4, *P* = 0.63, *I*^2^ = 0%). Effect measure: MD (original units); lower values indicate better performance.

In the meta-analysis, the inclusion of one study (Bin et al., 2022) markedly increased heterogeneity in the escape latency metrics. This finding suggests that the study differed from the others in aspects such as test setup, or experimental conditions, which may have primarily affected escape latency. The MWM test has certain experimental limitations, which may interfere with the experimental analysis and could be a source of heterogeneity in meta-analysis. A notable limitation of the MWM test is that the aversive conditions induce fluctuating stress in the animals, which impacts their performance, resulting in a U-shaped curve. Both insufficient and excessive stress levels can negatively affect learning outcomes (Vorhees and Williams, 2014). Due to the varying number of trials or training days in different experimental studies, the level of stress experienced by the rats may differ, which could lead to discrepancies in how well the experimental results reflect learning performance. Low temperature significantly impaired all spatial performance metrics (Rauch et al., 1989), hindering an accurate reflection of the rats’ true spatial learning ability, which, in turn, impacted the experimental outcomes. In addition, the animals’ vision may also interfere with the analysis of the MWM test. Previous studies have indicated that a decline in vision of rats can impair their spatial learning ability in the water maze task (Prusky et al., 2000). These factors may influence the experimental outcomes and potentially contribute to heterogeneity. Consequently, after excluding this study, our revised meta-analysis demonstrated a marked reduction in heterogeneity, as illustrated in Figure 3.

**FIGURE 4:**
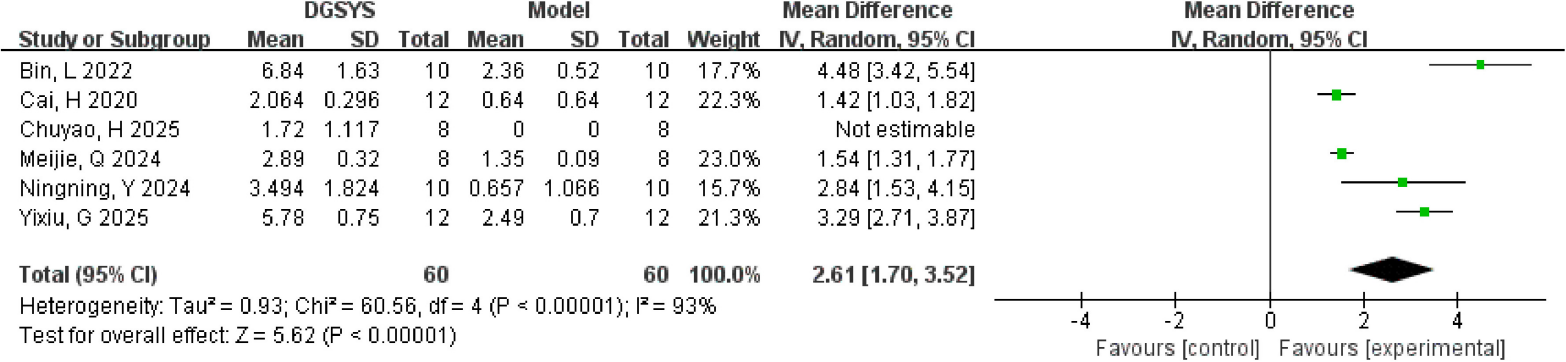
Forest plot of the number of platform crossings in the MWM test (DGSYS vs. control). Random-effects meta-analysis of 5 studies showed that DGSYS significantly increased the number of platform crossings compared with control (nT/nC = 52/52, MD = 2.61, 95% CI: 1.70–3.52, *P* < 0.00001; heterogeneity τ^2^ = 0.93, χ^2^ = 60.56, df = 4, *P* < 0.00001, *I*^2^ = 93%). One additional study was not estimable and therefore not included in the pooled estimate. Effect measure: MD (original units); higher values indicate better memory performance.

In addition, probe trial outcomes, such as platform crossings, were extracted when reported. Six studies reported the number of platform crossings as an outcome measure in the place navigation trial of the MWM test. Among them, three studies reported means and standard deviations, one study reported medians with interquartile ranges, and two studies (Cai et al., 2020; Ningning et al., 2024) presented graphical data that were extracted using WebPlotDigitizer version 4.7. We synthesized effects as mean differences (MDs) under a random-effects model comparing DGSYS with model controls, where positive MDs indicate more crossings in the DGSYS group. One study (Chuyao et al., 2025) was not estimable because the control group reported a standard deviation of zero, precluding inverse-variance pooling. This was consistent with the original report in which the interquartile range was zero, implying no within-group variability; therefore, this study was excluded from the meta-analysis for this outcome and summarized narratively. Meta-analysis of the remaining five studies indicated that DGSYS significantly increased the number of platform crossings compared to model controls [*n* = 104, MD = 2.61, 95% CI (1.70, 3.52), *P* < 0.00001] as shown in Figure 4. This represents a notable improvement in spatial memory performance in the DGSYS group. However, substantial heterogeneity was observed among the studies (Tau^2^ = 0.93, df = 4, *P* < 0.00001; *I*^2^ = 93%), indicating that the magnitude of DGSYS’s effect varied widely across different experiments.

**FIGURE 5:**
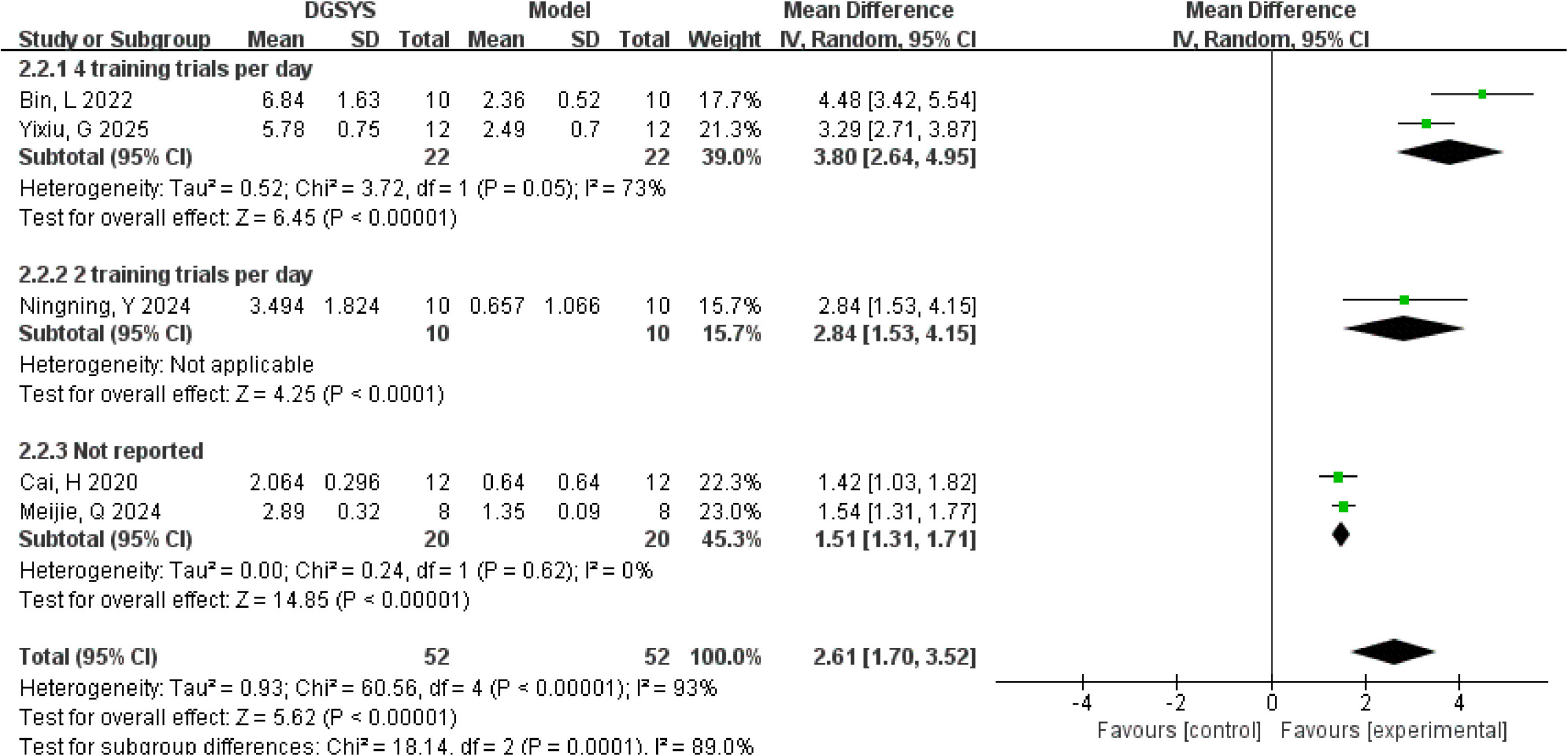
Subgroup forest plot of the number of platform crossings in the MWM test by training trials per day (DGSYS vs. control). Using a random-effects model, subgroup analysis showed that the effect differed by training trials per day. In the 4 training trials per day subgroup (2 studies; nT/nC = 22/22), DGSYS significantly increased the number of platform crossings (MD = 3.80, 95% CI: 2.64–4.95, *P* < 0.00001; heterogeneity τ^2^ = 0.52, χ^2^ = 3.72, df = 1, *P* = 0.05, *I*^2^ = 73%). In the 2 training trials per day subgroup (1 study; nT/nC = 10/10), DGSYS significantly increased the number of platform crossings (MD = 2.84, 95% CI: 1.53–4.15, *P* < 0.0001). In the not reported subgroup (2 studies; nT/nC = 20/20), DGSYS significantly increased the number of platform crossings (MD = 1.51, 95% CI: 1.31–1.71, *P* < 0.00001; heterogeneity τ^2^ = 0.00, χ^2^ = 0.24, df = 1, *P* = 0.62, *I*^2^ = 0%). A significant subgroup difference was observed (χ^2^ = 18.14, df = 2, *P* = 0.0001, *I*^2^ = 89.0%). Effect measure: MD (original units).

In subgroup analyses as shown in Figure 5 by training frequency, the effect was largest in studies with four training sessions per day [*n* = 44, MD = 3.80, 95% CI (2.64, 4.95), *P* < 0.00001; Tau^2^ = 0.52, df = 1; *I*^2^ = 73%]. The subgroup with two training sessions per day included one study and also favored DGSYS [*n* = 20, MD = 2.84, 95% CI (1.53, 4.15), *P* < 0.0001]. Studies with training frequency not reported showed a smaller but significant effect [*n* = 40, MD = 1.51, 95% CI (1.31, 1.71), *P* < 0.00001; Tau^2^ = 0.00, df = 1; *I*^2^ = 0%]. The difference between subgroups was significant (Chi^2^ = 18.14, df = 2, *P* = 0.0001). This suggests that training intensity may contribute to the observed heterogeneity.

The excluded study, which we summarized narratively, reported findings consistent with a beneficial effect of DGSYS. In that study, the DGSYS group showed a higher mean number of platform crossings (mean 1.72), whereas the model group showed no crossings on average (mean 0), consistent with better probe-trial performance. Although it could not be pooled because the control group reported a standard deviation of zero, the direction of effect is consistent with the overall meta-analytic finding that DGSYS improves spatial memory performance in vascular dementia models.

### 3.6 Effect of DGSYS on hippocampal neuronal morphology

Based on five experimental studies, DGSYS has been consistently shown to exert robust neuroprotective effects on hippocampal morphology in rat models of VaD. Morphological improvements were universally reported across studies, with DGSYS intervention leading to more clearly defined neuronal structures and restoration of hippocampal architecture (Meijie et al., 2024; Ningning et al., 2024). In particular, neurons within the CA1 region demonstrated markedly increased density and more orderly cellular arrangement (Cai et al., 2020; Meijie et al., 2024; Ningning et al., 2024), often approaching the histological appearance observed in sham-operated controls. Consistent with these findings, one study reported that neuronal morphology following high-dose DGSYS administration closely resembled that of sham-operated controls, however, the specific hippocampal subregion was not delineated (Yixiu et al., 2025). Notably, one study specifically examined the CA3 subregion and reported similar structural improvements, including reduced neuronal loss and promoted tighter cell alignment in this region (Chuyao et al., 2025). Moreover, three studies reported significant changes in hippocampal cell numbers, primarily characterized by increased neuronal density following DGSYS administration (Meijie et al., 2024; Chuyao et al., 2025; Yixiu et al., 2025).

### 3.7 Effect of DGSYS on TNF-α expression

**FIGURE 6:**
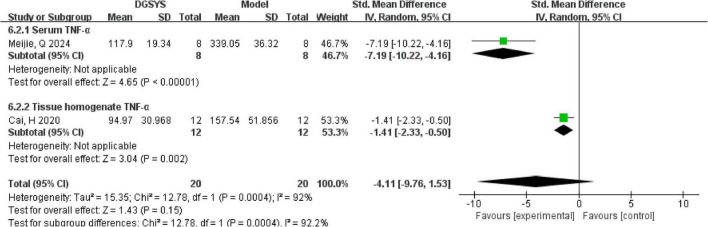
Subgroup forest plot of TNF-α expression (DGSYS vs. control). Using a random-effects model, subgroup analysis showed that the effect differed by sample type. In the serum TNF-α subgroup (1 study; nT/nC = 8/8), DGSYS significantly reduced TNF-α levels (SMD = –7.19, 95% CI: –10.22 to –4.16, *P* < 0.00001). In the tissue homogenate TNF-α subgroup (1 study; nT/nC = 12/12), DGSYS significantly reduced TNF-α levels (SMD = –1.41, 95% CI: –2.33 to –0.50, *P* = 0.002). A significant subgroup difference was observed (χ^2^ = 12.78, df = 1, *P* = 0.0004, *I*^2^ = 92.2%). Effect measure: SMD (standardized units); lower values indicate reduced TNF-α expression.

Two studies reported TNF-α levels as an outcome measure following DGSYS intervention. One study provided raw numerical data, while the other reported results graphically and the data were extracted using WebPlotDigitizer. We assessed the effect of DGSYS on TNF-α using standardized mean differences (SMDs) under a random-effects model. As shown in Figure 6, because TNF-α was measured in different biological matrices (serum vs. tissue homogenate), these results were considered not directly comparable and were therefore not pooled. In the serum assay, DGSYS significantly reduced TNF-α compared with model controls [*n* = 16, SMD = –7.19, 95% CI (–10.22, –4.16), *P* < 0.00001]. In the tissue homogenate assay, DGSYS also significantly decreased TNF-α [*n* = 24, SMD = –1.41, 95% CI (–2.33, –0.50), *P* = 0.002]. Both estimates favored DGSYS, but the magnitude of effect differed across matrices, supporting separate reporting rather than quantitative synthesis.

### 3.8 Effect of DGSYS on apoptosis regulation

**FIGURE 7:**
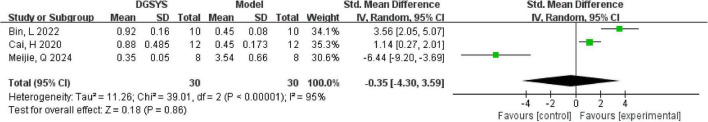
Forest plot of the Bcl-2/Bax ratio (DGSYS vs. control). Random-effects meta-analysis of 3 studies showed no significant difference in the Bcl-2/Bax ratio between groups (nT/nC = 30/30, SMD = –0.35, 95% CI: –4.30 to 3.59, *P* = 0.86; heterogeneity τ^2^ = 11.26, χ^2^ = 39.01, df = 2, *P* < 0.00001, *I*^2^ = 95%). Effect measure: SMD (standardized units).

Meta-analysis of three studies demonstrated that significant discrepancies in the effects of DGSYS on the Bcl-2/Bax ratio in rats with vascular dementia [*n* = 60, SMD = –0.35, 95% CI (–4.30, 3.59), *P* = 0.86] as shown in Figure 7. Two studies (Cai et al., 2020; Bin et al., 2022) reported a significant increase in the Bcl-2/Bax ratio following DGSYS administration, suggesting that DGSYS exerts neuroprotective effects by inhibiting neuronal apoptosis. In contrast, one study (Meijie et al., 2024) observed a reduction in the Bcl-2/Bax ratio after DGSYS treatment. This directional divergence resulted in substantial heterogeneity within the meta-analysis (*I*^2^ = 96%) and a non-significant pooled effect size. Possible sources of heterogeneity include differences in protein detection methods (Western blot vs. immunohistochemistry), variation in baseline references (normal control vs. model group), and statistical outliers in the model group. The divergence may be attributed to methodological differences in protein detection techniques (Western blot vs. immunohistochemistry), variability in reference baselines (normal control vs. model group), and data outliers within model groups.

**FIGURE 8:**
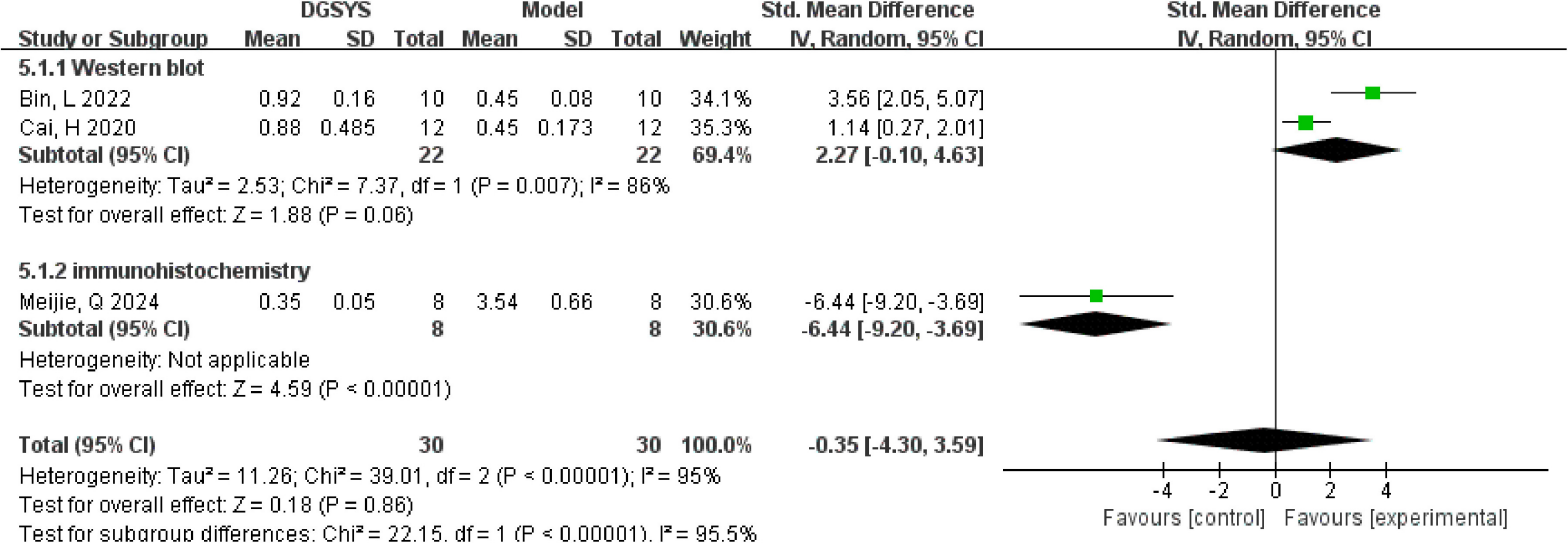
Subgroup forest plot of the Bcl-2/Bax ratio by detection method (DGSYS vs. control). Using a random-effects model, subgroup analysis showed that the effect differed by detection method. In the Western blot subgroup (2 studies; nT/nC = 22/22), DGSYS showed no significant change in the Bcl-2/Bax ratio compared with control (SMD = 2.27, 95% CI: –0.10 to 4.63, *P* = 0.06; heterogeneity τ^2^ = 2.53, χ^2^ = 7.37, df = 1, *P* = 0.007, *I*^2^ = 86%). In the immunohistochemistry subgroup (1 study; nT/nC = 8/8), the effect favored control (SMD = –6.44, 95% CI: –9.20 to –3.69, *P* < 0.00001). A significant subgroup difference was observed (χ^2^ = 22.15, df = 1, *P* < 0.00001, *I*^2^ = 95.5%). Effect measure: SMD (standardized units).

Given that the Bcl-2/Bax ratio was quantified using different detection methods (Western blot vs. immunohistochemistry), we performed a prespecified subgroup analysis by assay method as shown in Figure 8. In the Western blot subgroup, the pooled effect was not statistically significant [*n* = 44, SMD = 2.27, 95% CI (–0.10, 4.63), *P* = 0.06], with considerable heterogeneity (Tau^2^ = 2.53; Chi^2^ = 7.37, df = 1, *P* = 0.007; *I*^2^ = 86%). In contrast, the immunohistochemistry subgroup (single study) showed a significant decrease in the Bcl-2/Bax ratio after DGSYS treatment [*n* = 16, SMD = –6.44, 95% CI (–9.20, –3.69), *P* < 0.00001]. The difference between subgroups was significant (Chi^2^ = 22.15, df = 1, *P* < 0.00001; *I*^2^ = 95.5%), indicating that detection method may be a major source of the observed inconsistency. Therefore, current evidence does not support a consistent effect of DGSYS on the Bcl-2/Bax ratio. Future studies should standardize measurement approaches and reporting to improve comparability and reduce heterogeneity.

### 3.9 Effect of DGSYS on oxidative stress

According to meta-analysis of three studies, DGSYS exhibited significant effects on oxidative stress-related indicators in animal models of VaD.

**FIGURE 9:**
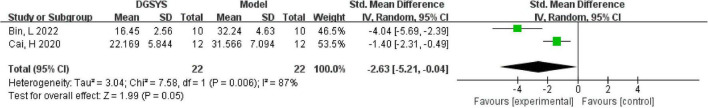
Forest plot of ROS levels (DGSYS vs. control). Random-effects meta-analysis of 2 studies showed that DGSYS significantly reduced ROS levels compared with control (nT/nC = 22/22, SMD = –2.63, 95% CI: –5.21 to –0.04, *P* = 0.05; heterogeneity τ^2^ = 3.04, χ^2^ = 7.58, df = 1, *P* = 0.006, *I*^2^ = 87%). Effect measure: SMD (standardized units); lower values indicate lower ROS levels.

**FIGURE 10:**
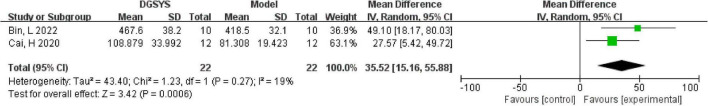
Forest plot of SOD levels (DGSYS vs. control). Random-effects meta-analysis of 2 studies showed that DGSYS significantly increased SOD levels compared with control (nT/nC = 22/22, MD = 35.52, 95% CI: 15.16–55.88, *P* = 0.0006; heterogeneity τ^2^ = 43.40, χ^2^ = 1.23, df = 1, *P* = 0.27, *I*^2^ = 19%). Effect measure: MD (original units); higher values indicate higher SOD levels.

**FIGURE 11:**
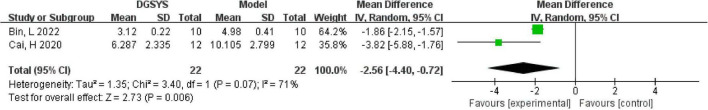
Forest plot of MDA levels (DGSYS vs. control). Random-effects meta-analysis of 2 studies showed that DGSYS significantly reduced MDA levels compared with control (nT/nC = 22/22, MD = –2.56, 95% CI: –4.40 to –0.72, *P* = 0.006; heterogeneity τ^2^ = 1.35, χ^2^ = 3.40, df = 1, *P* = 0.07, *I*^2^ = 71%). Effect measure: MD (original units); lower values indicate lower MDA levels.

Two studies assessed oxidative stress–related outcomes. As shown in Figure 9, DGSYS reduced ROS levels compared with model controls [*n* = 44, SMD = -2.63, 95% CI (–5.21, –0.04), *P* = 0.05], with considerable heterogeneity (Tau^2^ = 3.04, df = 1; *I*^2^ = 87%). As shown in Figure 10, DGSYS also increased SOD activity [*n* = 44, MD = 35.52, 95% CI (15.16, 55.88), *P* = 0.0006], with low heterogeneity (Tau^2^ = 43.40, df = 1; *I*^2^ = 19%). In addition, DGSYS significantly decreased malondialdehyde (MDA) levels [*n* = 44, MD = –2.56, 95% CI (–4.40, –0.72), *P* = 0.006], although heterogeneity was substantial (Tau^2^ = 1.35, df = 1; *I*^2^ = 71%) as shown in Figure 11. Substantial heterogeneity was observed for ROS and MDA, and the potential methodological sources of this variability are discussed below.

Collectively, these findings suggest that DGSYS may exert multifaceted protective effects against VaD by enhancing antioxidant enzyme activity, suppressing free radical production, and attenuating membrane lipid peroxidation. This provides experimental evidence supporting the therapeutic potential of DGSYS in oxidative stress-related neurodegenerative conditions.

## 4 Discussion

### 4.1 Summary of evidence

This meta-analysis synthesized preclinical evidence supporting the therapeutic potential of DGSYS in animal models of VaD. The pooled data showed that DGSYS significantly ameliorated behavioral impairments. Specifically, escape latency in the MWM test was markedly reduced [MD = –27.21, 95% CI (–30.36, –24.05), *P <* 0.0001], while the number of platform crossings was significantly increased [MD = 2.61, 95% CI (1.70, 3.52), *P <* 0.00001]. Besides, DGSYS markedly reduced oxidative stress markers, including MDA [MD = –2.56, 95% CI (–4.40, –0.72), *P* = 0.006]. Additionally, DGSYS was associated with increased Bcl-2 expression, an apoptosis-related marker, with the hippocampus exhibiting heightened sensitivity to this process. Because mechanistic endpoints were reported heterogeneously across studies, mechanistic interpretations should be considered supportive and largely qualitative rather than uniformly confirmed by pooled meta-analytic estimates. These multifaceted mechanisms position DGSYS as a promising candidate for targeting the complex pathological processes underlying VaD.

### 4.2 Exploration of mechanisms

DGSYS may exert therapeutic effects on VaD through multi-target synergistic regulation and the enhancement of neurovascular function (Kim and Cho, 2020). Emerging evidence suggests that DGSYS can ameliorate VaD through several mechanisms, including antioxidation, anti-inflammation, neuronal protection, and modulation of protein phosphorylation (Iadecola, 2013; Venkat et al., 2015; Wang et al., 2020); however, the precise pathways involved remain unclear. In this section, we distinguish outcomes supported by pooled analyses where available from mechanistic signals reported in individual studies and the broader literature, which should be interpreted as biologically plausible but not definitively established. Experimental studies suggest that DGSYS may provide neuroprotection by reducing inflammatory cytokine levels, alleviating oxidative stress, and protecting neurons from damage (Lu, 2001; Lan et al., 2012; Liu et al., 2012). These effects may involve the regulation of multiple signaling pathways, including LRP1 and LEP-R/GSK-3β/Tau (Cai et al., 2020; Bin et al., 2022; Ding et al., 2022). Nonetheless, the underlying mechanisms are complex and remain under ongoing debate, highlighting the need for further research to clarify the specific molecular interactions and causal relationships.

#### 4.2.1 Antioxidant activity

DGSYS may protect neurons from damage by reducing oxidative stress. Studies have shown that DGSYS significantly reduces levels of MDA and ROS, while enhancing SOD activity, thereby mitigating ischemia-reperfusion injury (Bin et al., 2022). Its antioxidative effects may be mediated, at least in part, by inhibiting the IKK/NF-κB signaling pathway, thereby indirectly suppressing the downstream effects of oxidative stress (Cai et al., 2020). Furthermore, DGSYS has been shown to attenuate oxidative and nitrosative stress, as well as inhibit neuronal apoptosis in the context of cerebral ischemia-reperfusion injury, via a SIRT1-dependent mechanism (Luo et al., 2021). In our meta-analysis, heterogeneity was substantial for ROS and MDA, which may be attributable to between-study differences in animal characteristics (Ruszkiewicz et al., 2019; McGrath et al., 2020), brain region analyzed (hippocampus vs. cortex) (Candelario-Jalil et al., 2001), assay methodology for oxidative-stress biomarkers (Kalyanaraman et al., 2012), and post-ischemic sampling time points (Kapoor et al., 2019).

#### 4.2.2 Anti-inflammatory activity

DGSYS alleviates neuroinflammation by suppressing pro-inflammatory cytokines and regulating key signaling pathways. It significantly reduces the expression of tumor necrosis factor-α (TNF-α) and interleukin-1β (IL-1β), both of which play key roles in exacerbating neuroinflammation in (VaD) (He et al., 2023). DGSYS upregulates low-density lipoprotein receptor-related protein 1 (LRP1), inhibiting the IKK/NF-κB signaling pathway and reducing the production of TNF-α and IL-1β (Cai et al., 2020). Additionally, DGSYS reduces the expression of the receptor for advanced glycation end products (RAGE) mRNA, a key receptor involved in inflammatory and oxidative stress responses (Meijie et al., 2024). Furthermore, DGSYS may mitigate neuroinflammation by suppressing microglial activation in the brain, limiting the release of IL-1β and TNF-α (Ou et al., 2023). Because these inflammatory endpoints were reported in a limited and heterogeneous manner, they should be interpreted as study-level mechanistic signals rather than pooled meta-analytic effects.

#### 4.2.3 Neuroprotection and anti-apoptosis

DGSYS protects neurons by reducing damage and apoptosis through multiple pathways. It stabilizes AMPA receptors and maintains GluR2 mRNA expression, reducing calcium permeability, preventing calcium overload, and mitigating excitotoxicity (Pu et al., 2005). Additionally, DGSYS suppresses the intrinsic apoptotic pathway by increasing the expression level of Bcl-2 and decreasing cleaved Caspase-3 levels, thus protecting hippocampal neurons from programmed cell death (Bin et al., 2022). DGSYS also enhances neuroprotection through the HIF-1α/EPO axis. Under hypoxic conditions, HIF-1α upregulates erythropoietin (EPO), which promotes neurogenesis and reduces neuronal apoptosis, improving cognitive function (Ningning et al., 2024). Moreover, DGSYS activates the PI3K/Akt signaling pathway by upregulating miR-124, promoting neuronal survival, and reducing hippocampal cell injury (Liu et al., 2024). However, apoptosis-related outcomes were not uniformly measured across studies, limiting quantitative confirmation.

#### 4.2.4 Protein and signaling pathway regulation

DGSYS regulates protein function via multiple signaling pathways. It enhances the clearance of amyloid-β (Aβ) by upregulating low-density lipoprotein receptor-related protein 1 (LRP1), reducing neuroinflammation and neurodegenerative changes (Meijie et al., 2024). Additionally, DGSYS increases the expression of leptin receptor (LEP-R) and phosphorylated glycogen synthase kinase-3β (p-GSK-3β), reducing phosphorylation of Tau protein at Ser404 and Ser202, which lowers abnormal Tau aggregation—an important feature in dementia pathology (Bin et al., 2022). Preliminary evidence suggests that DGSYS may modulate NMDA receptor activity and hippocampal glutamate levels, contributing to synaptic stabilization and preventing excitotoxicity (Yaozong and Bin, 2024). These pathway-level findings are hypothesis-generating and require further standardized replication.

### 4.3 Limitations

First, the literature search was limited to Chinese and English databases, which may introduce language bias and result in the omission of key studies published in other languages, affecting the completeness of the evidence base. Second, negative findings are less likely to be published. In this study, some original studies did not provide raw data, and certain information had to be estimated. The tendency for positive results to be preferentially published may overstate the true effect size and compromise the accuracy of effect estimates. Third, the quality scores of the included studies ranged from 4 to 6, with an average of 5.125, reflecting a moderate overall quality level. Therefore, caution is needed when interpreting the findings. Many preclinical studies are conducted with small sample sizes, and key safeguards such as rigorous randomization and blinding are not consistently implemented or clearly reported. Inadequate blinding may inflate effect estimates through performance bias and detection bias, thus weakening internal validity. Moreover, potential toxicity could not be evaluated because safety related outcomes were not reported in the included animal studies. Dose dependence was insufficiently examined, as dosing regimens varied substantially across studies, limiting inference on an optimal dose range. Furthermore, the quality standardization of herbal components was rarely described, which may reduce reproducibility and complicate cross study comparisons. Fourth, variations in modeling methods and animal species across studies may contribute to heterogeneity and confound the integration of results, representing a key source of variability in preclinical animal research. Therefore, the conclusions should be interpreted cautiously and further confirmed by larger, better designed, and more rigorously blinded experiments.

### 4.4 Clinical implications and future directions

Clinically, VaD remains an area of substantial unmet need because there are no licensed disease-modifying therapies, and currently available pharmacological options are largely symptomatic with at best modest efficacy (O’Brien and Thomas, 2015). Moreover, VaD is etiologically and lesion-wise heterogeneous, and concomitant Alzheimer-type pathology is common, which can complicate treatment-effect estimation and inform participant phenotyping and eligibility criteria in clinical trials (Ng et al., 2025). These clinical realities underscore the need to translate mechanistically grounded preclinical evidence into well-designed clinical evaluation.

Importantly, DGSYS has already been evaluated in human studies of dementia and has been summarized in a systematic review (Kim and Cho, 2020), which included studies in patients with vascular dementia. However, the available clinical evidence remains limited in scope and is heterogeneous in populations, formulations, comparators, and outcome reporting. These limitations restrict firm conclusions on efficacy and safety and highlight the need for better-designed clinical trials informed by intervention-specific preclinical evidence.

Within this clinical context, the present meta-analysis indicates that DGSYS shows potential translational value and biological plausibility in managing VaD, owing to its diverse and multi-targeted mechanisms of action. Reported effects include attenuation of oxidative stress and ischemia-related neuronal injury (Lan et al., 2012), as well as anti-inflammatory actions such as NF-κB-associated signaling and pro-inflammatory cytokine downregulation that may mitigate neuroinflammation and support cognition in VaD models (Cai et al., 2020). Beyond these mechanisms, DGSYS has also been shown to promote neuroprotection through the activation of several key signaling pathways, including HIF-1α/EPO, miR-124/PI3K/Akt, which may collectively reduce neuronal apoptosis and enhance learning and memory performance in animal studies (Chuyao et al., 2025). Furthermore, DGSYS appears to modulate hallmark pathological features of VaD by facilitating the clearance of Aβ via LRP1 upregulation and reducing Tau protein phosphorylation through the LEP-R/GSK-3β/Tau axis (Bin et al., 2022). Early evidence also points to its potential involvement in regulating cognitive function through the gut–brain axis, which represents an emerging direction for VaD research rather than an established therapeutic mechanism (He et al., 2023). Importantly, these findings should be interpreted cautiously: animal models do not fully capture the clinical heterogeneity of VaD, including mixed pathology, multimorbidity, polypharmacy, and variable treatment responsiveness.

In clinical settings, DGSYS is increasingly being recognized as a potential adjunct therapeutic option for VaD, particularly in light of the limited success of existing pharmacological interventions (Bai and Zhang, 2021). Preclinical studies have demonstrated that DGSYS may enhance various domains of cognitive performance, including memory, executive function, and daily living capabilities, suggesting translational relevance for patient-centered outcomes (Liu et al., 2024). As a traditional Chinese herbal formula, DGSYS is supported by a history of traditional use and is often considered tolerable; however, systematic evidence on long-term safety and herb–drug interactions in older VaD populations remains limited and requires prospective evaluation (Kim and Cho, 2020). Nonetheless, it is important to interpret these findings with caution, as significant differences exist between animal models and human VaD pathology. These include variability in disease etiology, the presence of comorbid conditions, and differences in treatment response, all of which may affect the clinical applicability of preclinical data.

Although DGSYS has demonstrated potential benefits in preclinical models of VaD (Ningning et al., 2024), several critical challenges remain before these findings can be effectively translated into clinical practice. One major limitation lies in the heterogeneity of existing animal models, including methods such as bilateral common carotid artery occlusion and ischemia-reperfusion injury, which contribute to inconsistencies in experimental outcomes and limit cross-study comparability (Washida et al., 2019). To facilitate clinical translation, future studies should prespecify dosing windows, treatment duration, and harmonized outcome measures, thereby improving comparability and strengthening the evidence base for clinical evaluation (Percie du Sert et al., 2020). Future work should also strengthen quality control of DGSYS and validate key mechanistic targets to inform trial design and endpoint selection (Chen et al., 2009; Lu et al., 2020).

Building on these prerequisites, the next step is rigorous clinical evaluation. Future studies should prioritize preregistered, adequately powered randomized controlled trials with appropriate comparators and sufficient follow-up to assess sustained effects. Clinically meaningful primary endpoints should include validated cognitive measures and functional outcomes, whereas biomarkers and neuroimaging measures can be incorporated as secondary or exploratory endpoints. Given the heterogeneity of VaD and the frequent presence of mixed pathology, prespecified phenotyping and stratification strategies are recommended to improve the interpretability of treatment effects.

Given its broad pharmacological profile, DGSYS may be considered for combination strategies with treatments used in VaD-related care (e.g., memantine or nimodipine); however, such potential synergy remains hypothesis-generating and should be tested empirically with careful safety monitoring (Wilcock et al., 2002; Pantoni et al., 2005; Li S. et al., 2022). Exploring combination therapy strategies could reveal additive or complementary benefits, potentially enhancing cognitive outcomes and slowing the progression of disease. At the same time, the marked interindividual variability observed among VaD patients underscores the importance of developing personalized treatment approaches (O’Brien and Thomas, 2015). Biomarker-based stratification—using indicators such as inflammatory cytokines or Tau protein levels—may help identify patient subgroups most likely to respond to DGSYS therapy (Zuliani et al., 2007; Skillbäck et al., 2015). Such a precision medicine framework would not only improve treatment efficacy but also facilitate more targeted and rational clinical application of DGSYS in the management of VaD. Such approaches should be treated as exploratory until validated in VaD-focused cohorts and trial settings.

DGSYS has demonstrated promise in modulating the gut-brain axis in models of AD (He et al., 2023); however, its involvement in VaD remains insufficiently explored (Liu et al., 2022). Given the growing recognition of the gut microbiome’s role in neurodegeneration, future research should prioritize both preclinical and clinical investigations to determine whether DGSYS can exert similar modulatory effects in VaD. Specifically, studies are needed to evaluate whether DGSYS promotes beneficial microbial populations while suppressing pro-inflammatory taxa, thereby contributing to cognitive improvement (Li et al., 2018). Further inquiry should also examine whether DGSYS-derived metabolites, or microbial transformation products, possess intrinsic neuroprotective properties (Loh et al., 2024). Clarifying these gut-mediated mechanisms may open new avenues for the development of microbiota-targeted interventions and broaden the therapeutic landscape of DGSYS in VaD treatment.

Although DGSYS is generally regarded as safe based on its long history of traditional use (Luo et al., 2024), concerns remain regarding potential risks associated with long-term administration, including gastrointestinal discomfort and possible herb–drug interactions. These issues are particularly relevant for elderly individuals with VaD, who frequently present with multiple comorbidities and are subject to polypharmacy.

To ensure comprehensive safety assessment, future research should incorporate long-term, follow-up-oriented study designs that actively monitor for adverse effects. Such efforts should include systematic tracking of adverse events, evaluation of pharmacokinetic interactions, and identification of cumulative or delayed toxicities related to chronic use (Sun et al., 2021). The resulting safety data will be critical for formulating evidence-based clinical guidelines on the use of DGSYS in patients with chronic neurodegenerative diseases.

In summary, the multi-target effects of DGSYS animal models of vascular dementia (VaD) provide a solid foundation for its potential clinical application. However, its efficacy and safety need further validation through high-quality clinical trials, in-depth mechanistic studies, and the development of standardized preparation methods. Future research should prioritize individualized treatment strategies, explore synergistic effects with existing therapies, and investigate the gut-brain axis as a novel mechanism of action. These efforts will be crucial for translating DGSYS from experimental evidence to practical application in the treatment of VaD.

## 5 Conclusion

Collectively, current preclinical evidence suggests the multifaceted therapeutic potential of DGSYS in treating VaD, with its multi-target actions that support further standardized preclinical validation. Across studies, the reported effects of DGSYS converge on several VaD-relevant themes, including oxidative stress and inflammatory regulation, neuroprotection and neurotransmission-related changes, and pathology-related readouts such as Aβ clearance and tau phosphorylation. These integrated actions suggest that these multi-pathway signals support the biological plausibility of DGSYS as a multi-target candidate for addressing the complex pathophysiology of VaD.

However, important barriers remain before these findings can be confidently extrapolated to clinical practice. Heterogeneity in animal models and protocols, small study sizes, and variable methodological rigor limit cross-study comparability and may inflate effect estimates, thereby constraining the translational value of the current preclinical evidence base.

A further limitation is that we extracted the highest-dose arm when multiple dose arms were reported, which may overestimate efficacy. Given the substantial between-study variability in dosing regimens and reporting, dose–response analyses were not feasible, and results should be interpreted cautiously. A further limitation is that we extracted the highest-dose arm when multiple dose arms were reported, which may overestimate efficacy. Given the substantial between-study variability in dosing regimens and reporting, dose–response analyses were not feasible, and results should be interpreted cautiously.

Notably, clinical studies of DGSYS in dementia, including vascular dementia, have already been systematically reviewed (Kim and Cho, 2020), but the available trials remain limited and do not address several internal-validity and mechanism-related questions that preclinical studies are positioned to inform.

Moving forward, preclinical studies should adopt prespecified and transparent design elements (randomization, blinding, sample-size estimation, and reporting of exclusions), harmonize key model parameters (model type, intervention window, treatment duration), and use a minimum core outcome set that includes standardized cognitive tests alongside histopathological and vascular readouts; in parallel, quality-controlled preparation and chemical characterization of DGSYS are needed to ensure batch-to-batch consistency and reproducibility across laboratories.

Overall, these steps support further standardized preclinical validation and may help guide the design of more rigorous future clinical investigations in VaD. Given substantial heterogeneity and limitations in risk of bias/reporting, conclusions should be interpreted cautiously.

## Data Availability

The original contributions presented in this study are included in this article/Supplementary material, further inquiries can be directed to the corresponding authors.

## Footnotes

1
https://automeris.io/WebPlotDigitizer
