# Supplementary material for: Efficacy of Dang Gui Shao Yao San in treating vascular dementia in animal models: a systematic review and meta-analysis
Source: Front Aging Neurosci. 2026 Mar 13;18:1701536. doi: 10.3389/fnagi.2026.1701536 (PMC13021646; doi:10.3389/fnagi.2026.1701536)
Supplement: Supplementary file 1 [file Data_Sheet_1.pdf]

Supplementary Table 1. Detailed Database Search Strategies

Database	Line	Search string
Supplementary Table 1. Detailed database search strategies for each database used in the review.		
CNKI (in Chinese)		TKA=('Danggui Shaoyao San (in Chinese)'+'Modified Danggui Shaoyao San (in Chinese)'+'Jiawei Danggui Shaoyao San (in Chinese)') AND TKA=('Vascular dementia (in Chinese)') AND FT=('Rat (in Chinese)'+'Mouse (in Chinese)')
Wanfang (in Chinese)		Topic (in Chinese):("Danggui Shaoyao San (in Chinese)"or "Modified Danggui Shaoyao San (in Chinese)"or"Jiawei Danggui Shaoyao San (in Chinese)") and Topic (in Chinese):("Vascular dementia (in Chinese)") and All (in Chinese):("Rat (in Chinese)"or"Mouse (in Chinese)")
VJIP Database (in Chinese)		(M=(Danggui Shaoyao San (in Chinese) or Modified Danggui Shaoyao San (in Chinese) or Jiawei Danggui Shaoyao San (in Chinese)) or K=(Danggui Shaoyao San (in Chinese) or Modified Danggui Shaoyao San (in Chinese) or Jiawei Danggui Shaoyao San (in Chinese))) and (M=Vascular dementia (in Chinese) or K=Vascular dementia (in Chinese)) and U=(Rat (in Chinese) or Mouse (in Chinese))
PubMed	#1	"danggui-shaoyao-san" [Supplementary Concept]
PubMed	#2	(danggui-shaoyao-san[Title/Abstract]) OR (danggui-jakyak-san[Title/Abstract])
PubMed	#3	((danggui-shaoyao-san[Title/Abstract]) OR (danggui-jakyak-san[Title/Abstract])) OR ("danggui-shaoyao-san" [Supplementary Concept])
PubMed	#4	"Dementia, Vascular"[Mesh]
PubMed	#5	((Dementia, Vascular[Title/Abstract]) OR (Dementias, Vascula[Title/Abstract])) OR (Vascular Dementias[Title/Abstract])) OR (Vascular Dementia[Title/Abstract])) OR (Arteriosclerotic Dementia[Title/Abstract])) OR (Arteriosclerotic Dementias[Title/Abstract])) OR (Dementia, Arteriosclerotic[Title/Abstract])) OR

		(Dementias, Arteriosclerotic[Title/Abstract])) OR (Binswanger Disease[Title/Abstract])) OR (Disease, Binswanger[Title/Abstract])) OR (Encephalopathy, Binswanger[Title/Abstract])) OR (Chronic Progressive Subcortical Encephalopathy[Title/Abstract])) OR (Binswanger Encephalopathy[Title/Abstract])) OR (Leukoencephalopathy, Subcortical[Title/Abstract])) OR (Leukoencephalopathies, Subcortical[Title/Abstract])) OR (Subcortical Leukoencephalopathies[Title/Abstract])) OR (Encephalopathy, Subcortical Arteriosclerotic[Title/Abstract])) OR (Encephalopathy, Chronic Progressive Subcortical[Title/Abstract])) OR (Encephalopathy, Subcortical, Chronic Progressive[Title/Abstract])) OR (Subcortical Encephalopathy, Chronic Progressive[Title/Abstract])) OR (Subcortical Leukoencephalopathy[Title/Abstract])) OR (Subcortical Arteriosclerotic Encephalopathy[Title/Abstract])) OR (Arteriosclerotic Encephalopathy, Subcortical[Title/Abstract])) OR (Arteriosclerotic Encephalopathies, Subcortical[Title/Abstract])) OR (Encephalopathies, Subcortical Arteriosclerotic[Title/Abstract])) OR (Subcortical Arteriosclerotic Encephalopathies[Title/Abstract])) OR (Encephalopathy, Binswanger's[Title/Abstract])) OR (Binswanger's Encephalopathy[Title/Abstract])) OR (Encephalopathy, Binswangers[Title/Abstract])) OR (Binswanger's Disease[Title/Abstract])) OR (Binswangers Disease[Title/Abstract])) OR (Disease, Binswanger's[Title/Abstract])) OR (Subcortical Vascular Dementia[Title/Abstract])) OR (Dementias, Subcortical Vascular[Title/Abstract])) OR (Dementia, Subcortical Vascular[Title/Abstract])) OR (Subcortical Vascular Dementias[Title/Abstract])) OR

		(Vascular Dementias, Subcortical[Title/Abstract])) OR (Vascular Dementia, Subcortical[Title/Abstract])) OR (Vascular Dementia, Acute Onset[Title/Abstract])) OR (Acute Onset Vascular Dementia[Title/Abstract])
PubMed	#6	(((Dementia, Vascular[Title/Abstract]) OR (Dementias, Vascula[Title/Abstract])) OR (Vascular Dementias[Title/Abstract])) OR (Vascular Dementia[Title/Abstract])) OR (Arteriosclerotic Dementia[Title/Abstract])) OR (Arteriosclerotic Dementias[Title/Abstract])) OR (Dementia, Arteriosclerotic[Title/Abstract])) OR (Dementias, Arteriosclerotic[Title/Abstract])) OR (Binswanger Disease[Title/Abstract])) OR (Disease, Binswanger[Title/Abstract])) OR (Encephalopathy, Binswanger[Title/Abstract])) OR (Chronic Progressive Subcortical Encephalopathy[Title/Abstract])) OR (Binswanger Encephalopathy[Title/Abstract])) OR (Leukoencephalopathy, Subcortical[Title/Abstract])) OR (Leukoencephalopathies, Subcortical[Title/Abstract])) OR (Subcortical Leukoencephalopathies[Title/Abstract])) OR (Encephalopathy, Subcortical Arteriosclerotic[Title/Abstract])) OR (Encephalopathy, Chronic Progressive Subcortical[Title/Abstract])) OR (Encephalopathy, Subcortical, Chronic Progressive[Title/Abstract])) OR (Subcortical Encephalopathy, Chronic Progressive[Title/Abstract])) OR (Subcortical Leukoencephalopathy[Title/Abstract])) OR (Subcortical Arteriosclerotic Encephalopathy[Title/Abstract])) OR (Arteriosclerotic Encephalopathy, Subcortical[Title/Abstract])) OR (Arteriosclerotic Encephalopathies, Subcortical[Title/Abstract])) OR (Encephalopathies, Subcortical Arteriosclerotic[Title/Abstract])) OR (Subcortical Arteriosclerotic Encephalopathies[Title/Abstract])) OR

		(Encephalopathy, Binswanger's[Title/Abstract])) OR (Binswanger's Encephalopathy[Title/Abstract])) OR (Encephalopathy, Binswangers[Title/Abstract])) OR (Binswanger's Disease[Title/Abstract])) OR (Binswangers Disease[Title/Abstract])) OR (Disease, Binswanger's[Title/Abstract])) OR (Subcortical Vascular Dementia[Title/Abstract])) OR (Dementias, Subcortical Vascular[Title/Abstract])) OR (Dementia, Subcortical Vascular[Title/Abstract])) OR (Subcortical Vascular Dementias[Title/Abstract])) OR (Vascular Dementias, Subcortical[Title/Abstract])) OR (Vascular Dementia, Subcortical[Title/Abstract])) OR (Vascular Dementia, Acute Onset[Title/Abstract])) OR (Acute Onset Vascular Dementia[Title/Abstract])) OR ("Dementia, Vascular"[Mesh])
PubMed	#7	"Mice"[Mesh]
PubMed	#8	((((((((((((((((((mice[Title/Abstract]) OR (Mus[Title/Abstract])) OR (Mouse[Title/Abstract])) OR (Mice, Laboratory[Title/Abstract])) OR (Laboratory Mice[Title/Abstract])) OR (Mouse, Laboratory[Title/Abstract])) OR (Laboratory Mouse[Title/Abstract])) OR (Mouse, Swiss[Title/Abstract])) OR (Swiss Mouse[Title/Abstract])) OR (Swiss Mice[Title/Abstract])) OR (Mice, Swiss[Title/Abstract])) OR (Mus domesticus[Title/Abstract])) OR (Mus musculus domesticus[Title/Abstract])) OR (domesticus, Mus musculus[Title/Abstract])) OR (Mus musculus[Title/Abstract])) OR (Mouse, House[Title/Abstract])) OR (House Mouse[Title/Abstract])) OR (Mice, House[Title/Abstract])) OR (House Mice[Title/Abstract])
PubMed	#9	("Mice"[Mesh]) OR (((((((((((((((((((mice[Title/Abstract]) OR (Mus[Title/Abstract])) OR (Mouse[Title/Abstract])) OR (Mice, Laboratory[Title/Abstract])) OR (Laboratory Mice[Title/Abstract])) OR (Mouse, Laboratory[Title/Abstract])) OR (Laboratory Mouse[Title/Abstract])) OR

		(Mouse, Swiss[Title/Abstract])) OR (Swiss Mouse[Title/Abstract])) OR (Swiss Mice[Title/Abstract])) OR (Mice, Swiss[Title/Abstract])) OR (Mus domesticus[Title/Abstract])) OR (Mus musculus domesticus[Title/Abstract])) OR (domesticus, Mus musculus[Title/Abstract])) OR (Mus musculus[Title/Abstract])) OR (Mouse, House[Title/Abstract])) OR (House Mouse[Title/Abstract])) OR (Mice, House[Title/Abstract])) OR (House Mice[Title/Abstract]))
PubMed	#10	"Rats"[Mesh]
PubMed	#11	((((((((((((rats[Title/Abstract]) OR (Rat[Title/Abstract])) OR (Rattus[Title/Abstract])) OR (Rats, Laboratory[Title/Abstract])) OR (Laboratory Rat[Title/Abstract])) OR (Laboratory Rats[Title/Abstract])) OR (Rat, Laboratory[Title/Abstract])) OR (Rattus norvegicus[Title/Abstract])) OR (norvegicus, Rattus[Title/Abstract])) OR (Rats, Norway[Title/Abstract])) OR (Norway Rat[Title/Abstract])) OR (Norway Rats[Title/Abstract])) OR (Rat, Norway[Title/Abstract])
PubMed	#12	("Rats"[Mesh]) OR (((((((((((((rats[Title/Abstract]) OR (Rat[Title/Abstract])) OR (Rattus[Title/Abstract])) OR (Rats, Laboratory[Title/Abstract])) OR (Laboratory Rat[Title/Abstract])) OR (Laboratory Rats[Title/Abstract])) OR (Rat, Laboratory[Title/Abstract])) OR (Rattus norvegicus[Title/Abstract])) OR (norvegicus, Rattus[Title/Abstract])) OR (Rats, Norway[Title/Abstract])) OR (Norway Rat[Title/Abstract])) OR (Norway Rats[Title/Abstract])) OR (Rat, Norway[Title/Abstract]))
PubMed	#13	(("Mice"[Mesh]) OR ((((((((((((((((((mice[Title/Abstract]) OR (Mus[Title/Abstract])) OR (Mouse[Title/Abstract])) OR (Mice, Laboratory[Title/Abstract])) OR (Laboratory Mice[Title/Abstract])) OR (Mouse, Laboratory[Title/Abstract])) OR (Laboratory Mouse[Title/Abstract])) OR (Mouse, Swiss[Title/Abstract])) OR (Swiss

		Mouse[Title/Abstract])) OR (Swiss Mice[Title/Abstract])) OR (Mice, Swiss[Title/Abstract])) OR (Mus domesticus[Title/Abstract])) OR (Mus musculus domesticus[Title/Abstract])) OR (domesticus, Mus musculus[Title/Abstract])) OR (Mus musculus[Title/Abstract])) OR (Mouse, House[Title/Abstract])) OR (House Mouse[Title/Abstract])) OR (Mice, House[Title/Abstract])) OR (House Mice[Title/Abstract]))) OR (("Rats"[Mesh]) OR (((((((((((((rats[Title/Abstract]) OR (Rat[Title/Abstract])) OR (Rattus[Title/Abstract])) OR (Rats, Laboratory[Title/Abstract])) OR (Laboratory Rat[Title/Abstract])) OR (Laboratory Rats[Title/Abstract])) OR (Rat, Laboratory[Title/Abstract])) OR (Rattus norvegicus[Title/Abstract])) OR (norvegicus, Rattus[Title/Abstract])) OR (Rats, Norway[Title/Abstract])) OR (Norway Rat[Title/Abstract])) OR (Norway Rats[Title/Abstract])) OR (Rat, Norway[Title/Abstract])))
PubMed	#14	(((danggui-shaoyao-san[Title/Abstract]) OR (danggui-jakyak-san[Title/Abstract])) OR ("danggui-shaoyao-san" [Supplementary Concept])) AND (((Dementia, Vascular[Title/Abstract]) OR (Dementias, Vascula[Title/Abstract])) OR (Vascular Dementias[Title/Abstract])) OR (Vascular Dementia[Title/Abstract])) OR (Arteriosclerotic Dementia[Title/Abstract])) OR (Arteriosclerotic Dementias[Title/Abstract])) OR (Dementia, Arteriosclerotic[Title/Abstract])) OR (Dementias, Arteriosclerotic[Title/Abstract])) OR (Binswanger Disease[Title/Abstract])) OR (Disease, Binswanger[Title/Abstract])) OR (Encephalopathy, Binswanger[Title/Abstract])) OR (Chronic Progressive Subcortical Encephalopathy[Title/Abstract])) OR (Binswanger Encephalopathy[Title/Abstract])) OR (Leukoencephalopathy, Subcortical[Title/Abstract])) OR (Leukoencephalopathies,

		Subcortical[Title/Abstract])) OR (Subcortical Leukoencephalopathies[Title/Abstract])) OR (Encephalopathy, Subcortical Arteriosclerotic[Title/Abstract])) OR (Encephalopathy, Chronic Progressive Subcortical[Title/Abstract])) OR (Encephalopathy, Subcortical, Chronic Progressive[Title/Abstract])) OR (Subcortical Encephalopathy, Chronic Progressive[Title/Abstract])) OR (Subcortical Leukoencephalopathy[Title/Abstract])) OR (Subcortical Arteriosclerotic Encephalopathy[Title/Abstract])) OR (Arteriosclerotic Encephalopathy, Subcortical[Title/Abstract])) OR (Arteriosclerotic Encephalopathies, Subcortical[Title/Abstract])) OR (Encephalopathies, Subcortical Arteriosclerotic[Title/Abstract])) OR (Subcortical Arteriosclerotic Encephalopathies[Title/Abstract])) OR (Encephalopathy, Binswanger's[Title/Abstract])) OR (Binswanger's Encephalopathy[Title/Abstract])) OR (Encephalopathy, Binswangers[Title/Abstract])) OR (Binswanger's Disease[Title/Abstract])) OR (Binswangers Disease[Title/Abstract])) OR (Disease, Binswanger's[Title/Abstract])) OR (Subcortical Vascular Dementia[Title/Abstract])) OR (Dementias, Subcortical Vascular[Title/Abstract])) OR (Dementia, Subcortical Vascular[Title/Abstract])) OR (Subcortical Vascular Dementias[Title/Abstract])) OR (Vascular Dementias, Subcortical[Title/Abstract])) OR (Vascular Dementia, Subcortical[Title/Abstract])) OR (Vascular Dementia, Acute Onset[Title/Abstract])) OR (Acute Onset Vascular Dementia[Title/Abstract])) OR ("Dementia, Vascular"[Mesh]))
PubMed	#15	((((danggui-shaoyao-san[Title/Abstract]) OR (danggui-jakyak-san[Title/Abstract])) OR ("danggui-shaoyao-san" [Supplementary Concept])) AND (((Dementia, Vascular[Title/Abstract]) OR (Dementias,

		Vascula[Title/Abstract])) OR (Vascular Dementias[Title/Abstract])) OR (Vascular Dementia[Title/Abstract])) OR (Arteriosclerotic Dementia[Title/Abstract])) OR (Arteriosclerotic Dementias[Title/Abstract])) OR (Dementia, Arteriosclerotic[Title/Abstract])) OR (Dementias, Arteriosclerotic[Title/Abstract])) OR (Binswanger Disease[Title/Abstract])) OR (Disease, Binswanger[Title/Abstract])) OR (Encephalopathy, Binswanger[Title/Abstract])) OR (Chronic Progressive Subcortical Encephalopathy[Title/Abstract])) OR (Binswanger Encephalopathy[Title/Abstract])) OR (Leukoencephalopathy, Subcortical[Title/Abstract])) OR (Leukoencephalopathies, Subcortical[Title/Abstract])) OR (Subcortical Leukoencephalopathies[Title/Abstract])) OR (Encephalopathy, Subcortical Arteriosclerotic[Title/Abstract])) OR (Encephalopathy, Chronic Progressive Subcortical[Title/Abstract])) OR (Encephalopathy, Subcortical, Chronic Progressive[Title/Abstract])) OR (Subcortical Encephalopathy, Chronic Progressive[Title/Abstract])) OR (Subcortical Leukoencephalopathy[Title/Abstract])) OR (Subcortical Arteriosclerotic Encephalopathy[Title/Abstract])) OR (Arteriosclerotic Encephalopathy, Subcortical[Title/Abstract])) OR (Arteriosclerotic Encephalopathies, Subcortical[Title/Abstract])) OR (Encephalopathies, Subcortical Arteriosclerotic[Title/Abstract])) OR (Subcortical Arteriosclerotic Encephalopathies[Title/Abstract])) OR (Encephalopathy, Binswanger's[Title/Abstract])) OR (Binswanger's Encephalopathy[Title/Abstract])) OR (Encephalopathy, Binswangers[Title/Abstract])) OR (Binswanger's Disease[Title/Abstract])) OR (Binswangers Disease[Title/Abstract])) OR

		(Disease, Binswanger's[Title/Abstract])) OR (Subcortical Vascular Dementia[Title/Abstract])) OR (Dementias, Subcortical Vascular[Title/Abstract])) OR (Dementia, Subcortical Vascular[Title/Abstract])) OR (Subcortical Vascular Dementias[Title/Abstract])) OR (Vascular Dementias, Subcortical[Title/Abstract])) OR (Vascular Dementia, Subcortical[Title/Abstract])) OR (Vascular Dementia, Acute Onset[Title/Abstract])) OR (Acute Onset Vascular Dementia[Title/Abstract])) OR ("Dementia, Vascular"[Mesh]))) AND ((("Mice"[Mesh]) OR (((((((((((((((((mice[Title/Abstract]) OR (Mus[Title/Abstract])) OR (Mouse[Title/Abstract])) OR (Mice, Laboratory[Title/Abstract])) OR (Laboratory Mice[Title/Abstract])) OR (Mouse, Laboratory[Title/Abstract])) OR (Laboratory Mouse[Title/Abstract])) OR (Mouse, Swiss[Title/Abstract])) OR (Swiss Mouse[Title/Abstract])) OR (Swiss Mice[Title/Abstract])) OR (Mice, Swiss[Title/Abstract])) OR (Mus domesticus[Title/Abstract])) OR (Mus musculus domesticus[Title/Abstract])) OR (domesticus, Mus musculus[Title/Abstract])) OR (Mus musculus[Title/Abstract])) OR (Mouse, House[Title/Abstract])) OR (House Mouse[Title/Abstract])) OR (Mice, House[Title/Abstract])) OR (House Mice[Title/Abstract]))) OR (("Rats"[Mesh]) OR (((((((((((((rats[Title/Abstract]) OR (Rat[Title/Abstract])) OR (Rattus[Title/Abstract])) OR (Rats, Laboratory[Title/Abstract])) OR (Laboratory Rat[Title/Abstract])) OR (Laboratory Rats[Title/Abstract])) OR (Rat, Laboratory[Title/Abstract])) OR (Rattus norvegicus[Title/Abstract])) OR (norvegicus, Rattus[Title/Abstract])) OR (Rats, Norway[Title/Abstract])) OR (Norway Rat[Title/Abstract])) OR (Norway Rats[Title/Abstract])) OR (Rat, Norway[Title/Abstract]))))
SCI	#1	ALL= （mice OR Mus OR Mouse OR Mice, Laboratory OR Laboratory Mice OR

		Mouse, Laboratory OR Laboratory Mouse OR Mouse, Swiss OR Swiss Mouse OR Swiss Mice OR Mice, Swiss OR Mus domesticus OR Mus musculus domesticus OR domesticus, Mus musculus OR Mus musculus OR Mouse, House OR House Mouse OR Mice, House OR House Mice OR rats OR Rat OR Rattus OR Rats, Laboratory OR Laboratory Rat OR Laboratory Rats OR Rat, Laboratory OR Rattus norvegicus OR norvegicus, Rattus OR Rats, Norway OR Norway Rat OR Norway Rats OR Rat, Norway）
SCI	#2	TS=（'dementia, multi-infarct' OR 'dementia, multiinfarct' OR 'dementia, vascular' OR 'lacunar dementia' OR 'multi-infarct dementia' OR 'multi-infarction dementia' OR 'multiinfection dementia' OR 'vascular dementia' OR 'multiinfarct dementia'）
SCI	#3	TS=（'dang gui shao yao san' OR 'dang-gui-shao-yao-san' OR 'danggui shaoyao san' OR 'danggui-shaoyao-san' OR 'dangguishaoyaosan' OR 'tj 23' OR 'tj-23' OR 'tc0237' OR 'to23' OR 'toki shakuyaku san' OR 'toki-shakuyaku-san' OR 'tokishakuyaku san extract' OR 'tokishakuyaku-san' OR 'tokishakuyakusan' OR 'tokishakuyaku san'）
SCI	#4	#1 AND #2 AND #3
embase	#1	tokishakuyaku san'/exp OR 'tokishakuyaku san'
embase	#2	dang gui shao yao santab,ti OR 'danggui shaoyao san' ab,ti OR dangguishaoyaosan:ab,ti OR 'tj 23' ab、ti OR tj023e ab,ti OR tj23 ab,ti OR 'toki shakuyaku san' ab ti OR 'tokishakuyaku san extract' ab,ti OR tokishakuyakusan:ab,ti OR 'tokishakuyaku san' ab ti
embase	#3	#1 OR #2
embase	#4	multiinfarct dementia'/exp OR 'multiinfarct dementia'
embase	#5	dementia, multi-infarct' ab,ti OR ''dementia, vascular' ab ti OR 'lacunar dementia'tab,ti OR 'multi-infarct dementia' ab,ti OR 'multi-infarction dementia' ab,ti OR ''multiinfarction demential ab ti OR 'vascular dementia' ab ti OR 'multiinfarct dementia' ab,ti
embase	#6	#4 OR #5

embase	#7	#3 AND #6
sinomed	#1	"Danggui Shaoyao Tang (in Chinese)"[Unweighted (in Chinese):Expanded (in Chinese)]
sinomed	#2	"Dementia (in Chinese),Vascular (in Chinese)"[Unweighted (in Chinese):Expanded (in Chinese)]
sinomed	#3	"Rat (in Chinese)"[Unweighted (in Chinese):Expanded (in Chinese)]
sinomed	#4	"Mouse (in Chinese)"[Unweighted (in Chinese):Expanded (in Chinese)]
sinomed	#5	"Danggui Shaoyao San (in Chinese)"[Common field (in Chinese):Smart (in Chinese)]
sinomed	#6	#1 OR #5
sinomed	#7	"Vascular dementia (in Chinese)"[Common field (in Chinese):Smart (in Chinese)]
sinomed	#8	#7 OR #2
sinomed	#9	"Rat (in Chinese)"[Common field (in Chinese):Smart (in Chinese)]
sinomed	#10	#9 OR #3
sinomed	#11	"Mouse (in Chinese)"[Common field (in Chinese):Smart (in Chinese)]
sinomed	#12	#11 OR #4
sinomed	#13	#12 OR #10
sinomed	#14	#8 AND #6
sinomed	#15	#13 AND #14
cochrane	#1	MeSH descriptor: [Dementia, Vascular] explode all trees
cochrane	#2	Binswanger Disease OR Subcortical Arteriosclerotic Encephalopathy OR Disease, Binswanger's OR Subcortical Arteriosclerotic Encephalopathies OR Binswanger Encephalopathy OR Subcortical Encephalopathies, Subcortical OR Leukoencephalopathy, Subcortical OR Binswanger's Disease OR Subcortical Leukoencephalopathies OR Subcortical Encephalopathy, Chronic Progressive OR Binswanger Disease OR Encephalopathy, Subcortical, Chronic Progressive OR Encephalopathy, Chronic Progressive Subcortical OR Dementia, Subcortical Vascular OR Vascular Dementia, Subcortical OR Vascular Dementias, Subcortical Vascular OR Dementias, Arteriosclerotic OR Arteriosclerotic Dementia OR Acute Onset Vascular Dementia OR Vascular Dementia, Acute Onset

cochrane	#3	#1 OR #2
cochrane	#4	"danggui shao yao san" OR "danggui-shaoyao-san"
cochrane	#5	#3 AND #4
